# The cytokine receptor CRLF3 is a human neuroprotective EV-3 (Epo) receptor

**DOI:** 10.3389/fnmol.2023.1154509

**Published:** 2023-04-06

**Authors:** Debbra Y. Knorr, Ignacio Rodriguez Polo, Hanna S. Pies, Nicola Schwedhelm-Domeyer, Stephanie Pauls, Rüdiger Behr, Ralf Heinrich

**Affiliations:** ^1^Department of Cellular Neurobiology, Johann-Friedrich-Blumenbach Institute for Zoology and Anthropology, Georg-August University Göttingen, Göttingen, Germany; ^2^Department of Developmental Biology, Göttingen Center for Molecular Biosciences, Georg-August University Göttingen, Göttingen, Germany; ^3^Research Platform Degenerative Diseases, German Primate Center, Leibniz Institute for Primate Research, Göttingen, Germany; ^4^German Center for Cardiovascular Research (DZHK), Partner Site Göttingen, Göttingen, Germany; ^5^Developmental Models Laboratory, The Francis Crick Institute, London, United Kingdom

**Keywords:** CRLF3, cytokine receptor, neuroprotection, erythropoietin, EPO, EV-3, apoptosis, IPSC

## Abstract

The evolutionary conserved orphan cytokine receptor-like factor 3 (CRLF3) has been implicated in human disease, vertebrate hematopoiesis and insect neuroprotection. While its specific functions are elusive, experimental evidence points toward a general role in cell homeostasis. Erythropoietin (Epo) is a major regulator of vertebrate hematopoiesis and a general cytoprotective cytokine. Erythropoietic functions mediated by classical Epo receptor are understood in great detail whereas Epo-mediated cytoprotective mechanisms are more complex due to involvement of additional Epo receptors and a non-erythropoietic splice variant with selectivity for certain receptors. In the present study, we show that the human CRLF3 mediates neuroprotection upon activation with the natural Epo splice variant EV-3. We generated *CRLF3* knock-out iPSC lines and differentiated them toward the neuronal lineage. While apoptotic death of rotenone-challenged wild type iPSC-derived neurons was prevented by EV-3, EV-3-mediated neuroprotection was absent in *CRLF3* knock-out neurons. Rotenone-induced apoptosis and EV-3-mediated neuroprotection were associated with differential expression of pro-and anti-apoptotic genes. Our data characterize human CRLF3 as a receptor involved in Epo-mediated neuroprotection and identify CRLF3 as the first known receptor for EV-3.

## Introduction

Various hematopoietic growth factors that regulate the production of blood cells mediate additional homeostatic functions in other non-hematopoietic tissues. Examples are erythropoietin (Epo), thrombopoietin (Tpo) and granulocyte colony-stimulating factor (GCSF), all of which are also expressed in mammalian nervous systems where they regulate development, differentiation and cell survival (Dame et al., 2003; Schäbitz et al., 2003; Schneider et al., 2005; Byts et al., 2006). Their receptors (EpoR, TpoR, and GCSFR) belong to the class I family of cytokine type I receptors that possess the extracellular cytokine receptor homology domain and an WSXWS motif (Boulay et al., 2003; Liongue and Ward, 2007; Hahn et al., 2019). Another member of this hematopoietic cytokine receptor family is the orphan cytokine receptor-like factor 3 (CRLF3) which was recently implicated in zebrafish hematopoiesis (Taznin et al., 2022) and mammalian thrombopoiesis (Bennett et al., 2022). Human CRLF3 contains the characteristic cytokine receptor motif (WSXWS), a single-pass transmembrane region and a Janus kinase docking site and is expressed in various tissues including the nervous system (Boulay et al., 2003; Hahn et al., 2017). Though its ligand has not been identified and signaling mechanisms are largely unknown, CRLF3 has been associated with the regulation of proliferation, differentiation and cell survival (Hashimoto et al., 2012; Wegscheid et al., 2021) similar to its structurally related receptors for other hematopoietic cytokines. Moreover, CRLF3 levels have been detected in tumors and various tumor cell lines (Dang et al., 2006; Yang et al., 2009) and sequence alterations have been linked to amyotrophic lateral sclerosis (ALS; Cirulli et al., 2015), autism spectrum disorders (Wegscheid et al., 2021) and sensitivity to *Leishmania* infections (Castellucci et al., 2021). In contrast to the vertebrate-specific members of its cytokine receptor family, CRLF3 is highly conserved and present in all major eumetazoan taxa including cnidarians, various invertebrates and vertebrates including humans (Boulay et al., 2003; Liongue and Ward, 2007; Hahn et al., 2019; Taznin et al., 2022). Previous studies on locusts and beetles reported that the insect ortholog of CRLF3 mediates potent neuroprotection upon stimulation with human recombinant Epo (Hahn et al., 2017, 2019).

The vertebrate-specific helical cytokine erythropoietin (Epo) is a kidney-derived hormonal regulator of hematopoiesis, that protects erythroid progenitor cells from apoptosis to promote increased numbers of oxygen-transporting mature erythrocytes (Jelkmann and Metzen, 1996; Constantinescu et al., 1999; Lundby and Olsen, 2011). Local expression and release of Epo has been described for various tissues including brain, liver and lung and numerous studies reported its cytoprotective and regenerative functions in these and other tissues [reviewed in Ghezzi and Brines (2004), Chateauvieux et al. (2011), and Zhang et al. (2014)]. With respect to the nervous system, Epo is crucial for normal brain development, acts neuroprotectively after hypoxic/ischemic and other toxic insults and promotes regeneration after axonal damage (Morishita et al., 1996; Yu et al., 2002; Genc et al., 2004; Kretz et al., 2005). Beneficial functions also included enhanced cognitive performance and memory functions in healthy humans and patients affected by schizophrenia and mood disorders (Miskowiak et al., 2007, 2008, 2016; Kästner et al., 2012). Clinical studies explored the potential of Epo to interfere with cell loss in neurodegenerative diseases including Alzheimer’s disease, Parkinson’s disease and amyotrophic lateral sclerosis (Maiese et al., 2008; Chong et al., 2013; Rey et al., 2019). A drawback of prolonged and/or high dose Epo administration is the overproduction of erythrocytes leading to increased risk of thrombosis, stimulation of cancerogenic cell proliferation and tumor vascularization all resulting from activation of homodimeric classical EpoR (Hardee et al., 2006; Ehrenreich et al., 2009; Pedroso et al., 2012; Cao, 2013). Studies with Epo-mimetics (some with partial sequence similarity, others with rather unrelated structure compared to full Epo) have demonstrated neuroprotective and regenerative effects without activation of homodimeric EpoR (Leist et al., 2004; Brines et al., 2008; Ueba et al., 2010; Wu et al., 2013). Some of these may activate a heteroreceptor consisting of EpoR and ß common receptor (synonym CD131), which mediates neuroprotection in some but not all brain regions (Brines et al., 2004; Nadam et al., 2007; Sanchez et al., 2009b; Chamorro et al., 2013; Miller et al., 2015; Ding et al., 2017). The naturally occurring Epo splice variant EV-3, characterized by the lack of exon 3, mediates neuroprotection independent of both homodimeric and heteromeric EpoR suggesting that additional alternative neuroprotective receptors for Epo-like signals exist in the mammalian brain (Bonnas et al., 2017; Ostrowski and Heinrich, 2018).

Insects and other invertebrates lack orthologues of vertebrate Epo and EpoR. Human recombinant Epo and EV-3 increased the survival of hypoxia-or toxin-challenged insect neurons by activation of cytokine receptor-like factor 3 (CRLF3; Miljus et al., 2014, 2017; Hahn et al., 2017, 2019) suggesting that CRLF3 might be a general neuro-or tissue-protective receptor for Epo-like cytokines across species. CRLF3 shares both structural similarity and regulatory roles in blood cell production with EpoR, TpoR, and GCSFR (Boulay et al., 2003; Liongue and Ward, 2007; Bennett et al., 2022; Taznin et al., 2022). All of these receptors are also expressed in vertebrate nervous systems but, in contrast to EpoR, TpoR, and GCSFR, neither the activating ligand nor a concrete function of CRLF3 in non-hematopoietic tissue were so far identified.

In light of highly conserved *CRLF3* orthologues in insects and mammals, we hypothesized that Epo mediates cell-protective functions *via* activation of CRLF3 in human neurons, as it was previously reported for insect CRLF3. To study whether Epo/CRLF3-signaling protects human neurons from stress-induced apoptosis we established survival assays with human induced pluripotent stem cell-derived neurons.

Induced pluripotent stem cells (iPSC), that can give rise to various cell types upon exposure to appropriate differentiation protocols, harbor great potential for biomedical research and disease modelling (Doss and Sachinidis, 2019; Rodriguez-Polo et al., 2019; Wiegand and Banerjee, 2019; Stauske et al., 2020). We generated *CRLF3* knock out (KO) iPSC lines along with isogenic control lines (Ig-Ctrl) from two independent human iPSC lines by means of a Piggy-Bac-CRISPR-Cas9 system and differentiated them into neuron-like cells. Apoptosis was induced through “chemical hypoxia” by addition of rotenone, an inhibitor of complex I of the mitochondrial electron transport chain. CRLF3 was stimulated with the natural human Epo splice variant EV-3 to prevent coactivation of homodimeric EpoR or EpoR/βcR (Bonnas et al., 2017). We demonstrate that EV-3 protects WT and Ig-Ctrl iPSC-derived neurons from rotenone-induced apoptosis. In contrast, *CRLF3*-KO neurons were not protected, indicating that CRLF3 serves as neuroprotective receptor for EV-3 in human neurons. Rotenone exposure and EV-3 treatment altered expression or activity level of pro- (BAX, Caspse 3) and anti- (BCL-2) apoptotic factors. The results of our study deorphanize human CRLF3 by identifying EV-3 (and also Epo) as a natural ligand. Moreover, we show that EV-3/CRLF3 signaling mediates protection of human cells indicating that CRLF3 can be selectively targeted by Epo-like ligands to counteract neurodegenerative diseases without simultaneously promoting inappropriate erythropoiesis and tumor growth.

## Methods

Experiments were conducted with two human iPSC lines. iPSC were generated from commercially available human fibroblasts originating from a female and a male patient (referred to from now as iPSC#1 and iPSC#2 respectively; Lonza CC-2511, lot 0000490824 [iPSC#1], and lot 0000545147 [iPSC#2]). Reprogramming was performed according to Okita et al. (2011). iPSC characterization was described in Stauske et al. (2020). Human iPSC were maintained at 37°C, 5% CO_2_ in Universal primate pluripotent stem cell medium (UPPS medium), and cell splitting was performed using Versene solution (Thermo Fisher Scientific; #15040066) according to Stauske et al. (2020). iPSC were cultured on Geltrex-coated 6 cm or 12-well dishes (Thermo Fisher; A1413202). Basic authentication by characterization of each iPSC line is performed routinely to guarantee the identity and pluripotent state.

For all molecular analysis described below, cells (both iPSC and iPSC-derived neurons) were washed twice in Phosphate buffered saline (PBS) before being scraped (Cell scraper, Sarstedt; #833945040) and collected in an 1.5 mL Eppendorf tube. Cell suspensions were centrifuged at 12,000 × g for 2 min. PBS was removed and cell pellets snap-frozen in liquid nitrogen. Samples were stored at −80°C until further analysis.

### Establishment and characterization of transgenic lines

The KO lines were generated according to Rodriguez-Polo et al. (2021), following a constitutive Cas9-gRNA expression strategy. In brief, cells were nucleofected with a piggyBac-CRISPR-Cas9-GFP vector carrying a guide RNA (gRNA) specifically targeting *CRLF3* (see Table 1) and a second vector carrying Transposase-dtTomato (Pac-PB-Tomato; Debowski et al., 2015). In parallel, a different subset of cells was transfected with an empty piggyBac-CRISPR-Cas9 (no gRNA) construct in order to generate isogenic control (Ig-Ctrl) lines. After transfection the GFP positive (Cas9-GFP-gRNA positive) population was sorted by Fluorescence assisted cell sorting (FACS; Sony Flow Cytometry FACS SH800S). The presence of INDEL mutations in the polyclonal population was evaluated using PCR (Primer sequences see Table 1) in combination with T7 endonuclease I assay and Sanger sequencing. For the generation of the CRLF3 KO monoclonal lines, polyclonal populations were single-cell sorted into a 96-well plate, expanded and genotyped. Presence of the transgene was evaluated by GFP expression using a Zeiss Observer Fluorescent microscope (Carl Zeiss, #4001584). Successful introduction of loss-of-function mutations was evaluated in the monoclonal lines amplifying the targeted locus by PCR, subcloning the product in pCRII vector (TA cloning kit; Thermo Fisher Scientific # K207020), transforming the vector into competent *E. coli*, and sequencing by Sanger (20 bacterial clones per cell line analyzed). Subsequent sequence analysis revealed allele-specific variations in each one of the iPSC clones. Additionally, protein depletion from mutated iPSC and iPSC-neurons was confirmed by Western blot (see below).

**Table 1:** Oligonucleotides.

	Gene	Oligonucleotide 5′–3′	Tm	Accession number
gRNA	CRLF3-fwd	CACCGAAAGGCCTCGCACATTCAGT	61°C	ENSP00000318804.6
gRNA	CRLF3-rev	AAACACTGAATGTGCGAGGCCTTTC		
PCR	CRLF3-fwd	CCCTGGGCTTTCTGCTTTGC		
PCR	CRLF3-rev	ACCACGCATGGTCTGAAAACC	61°C	
qPCR	CRLF3 fwd	CAACGTTGGGGTCTATGTGC		
qPCR	CRLF3 rev	CGCCCACCAGTACAGATAGA		
qPCR	Bax-fwd	CGAGTGGCAGCTGACATGTT	61°C	ENST00000293288.12
qPCR	Bax-rev	TCCAGCCCATGATGGTTCTG		
qPCR	Caspase 3-fwd	GGAGGCCGACTTCTTGTATG	61°C	ENST00000308394.9
qPCR	Caspase 3-rev	TGCCACCTTTCGGTTAACCC		
qPCR	BCL-2-fwd	CGTTATCCTGGATCCAGGTG	61°C	ENST00000398117.1
qPCR	BCL-2-rev	GTGTGTGGAGAGCGTCAAC		
qPCR	bActin-fwd	GCGAGAAGATGACCCAGATC	61°C	ENST00000674681.1
qPCR	bActin-rev	GGGCATACCCCTCGTAGATG		

#### Genomic DNA extraction and PCR

Genomic DNA (gDNA) was extracted using DNeasy Blood & Tissue Kit (Qiagen; #69504) according to the manufacturer’s instructions. The gRNA target site was amplified using specific primers as stated below (Table 1). PCR was run using GoTaq Green Master Mix (Promega; #M7122) and PCR program was set as shown in Table 2. PCR products were loaded on a 1% agarose gel and run for 30 min at 100 V before extracting DNA fragments using Macherey–Nagel NucleoSpin Gel and PCR Clean-up Kit (Macherey–Nagel; #740609.50). The isolated DNA fragments were subsequently either sent for sequencing using specific PCR primers (for Polyclonal approach; Sequencing facility Microsynth AG, Göttingen, Germany) or cloned into a pCRII vector for allele characterization (for clonal expansion).

**Table 2:** PCR program for CRLF3 amplification.

Step	Temperature [°C]	Time [s]	Cycle
Initial denaturation	95	180	×30
Denaturation	95	30	
Annealing	61	30	
Elongation	72	30	
Final elongation	72	300	

#### Transformation

pCRII vectors carrying PCR products of single-cell clones were transformed into XL1-blue competent cells (Agilent; #200249). 500 ng plasmid were carefully mixed with 100 μL of competent cells and let to rest on ice for 30 min. Subsequently, cells received a heat shock at 42°C for 40 s before 900 μL super optimal broth (SOB; Roth; #AE27.1) without antibiotics was added. Cell suspension was transferred into a bacterial incubator for 1 h at 37°C, 225 rpm. Afterwards, cell suspension was centrifuged at 3,000 × g for 2 min, supernatant was removed, and cells were resuspended in 100 mL SOB medium before being dispersed on LB agar plates + ampicillin (Sigma-Aldrich; #L2897). Plates were let to rest at room temperature (RT) for 10 min before being transferred to 37°C.

#### Western blot

Cell pellets were lysed in protein lysis buffer (150 mM NaCL; 20 mM Tris.HCl pH 7.5; 1 mM EDTA; 1% Triton-X-100) + Protease inhibitor (Thermo Fisher Scientific; #78429) by vigorous shaking in a tissue lyser (Qiagen; #85300) for 3 min at 50 Hz. Subsequently, the lysates were transferred onto ice and incubated for 30 min. Cell lysate was centrifuged at 10,000 × g for 10 min at 4°C and the protein containing supernatant was transferred to a fresh Eppendorf tube. Protein concentration was measured by Bradford assay (PanReac AppliChem; #A69320500). For all Western blots run in this study 50 μg protein (except 100 μg for cleaved caspase 3 and Epo detection) was denatured in 2X Lämmli buffer (Sigma-Aldrich; #S3401) at 95°C for 5 min. 10% SDS-Pages were run for 30 min at 70 V and 1 h at 120 V. For size reference, PageRuler Plus Prestained Protein ladder (Thermo Fisher Scientific; #26619) was loaded together with samples. The separated protein was transferred onto nitrocellulose membranes (Roth; #9200.1) in a wet blot approach for 1.5 h at 180 mA. Membranes were incubated in Ponceau S (Sigma-Aldrich; #P3504) in order to check for sufficient and successful protein transfer before being blocked in 5% Milk/PBS-0.1% Tween-20 (PBST) for 30 min at room temperature (Milk Roth; #T145; Tween-20 PanReac AppliChem; #A7564). Membranes were probed for CRLF3 (see antibody list for dilutions in Table 3) either at RT for 2 h or overnight at 4°C. Subsequently, membranes were washed 3 times in PBST before incubation in α-HRP solution for 30 min at RT. Membranes were imaged by incubation in Pierce ECL Western blotting substrate (Thermo Fisher Scientific; #32209) using iBright CL1500 Imaging System (Thermo Fisher Scientific; **#**A44114). Subsequently, membranes were stripped in 0.5 M NaOH for 3 min, washed 3 times in PBS before being blocked again. Membranes were incubated in αTubulin (see Table 3) for 1 h at RT before incubation with the secondary α-HRP antibody and imaging. Quantification of protein band intensities was performed using ImageJ. Band intensities were normalized to the corresponding αTubulin band intensity of each sample and then toward control samples within treatment groups. Data is shown as bar plots representing the average band intensities measured together with the calculated standard deviation and single data points.

**Table 3:** Antibodies used.

Antibody	Company	Host	Dilution	Application
CRLF3	Santa Cruz; #sc-398,388	Mouse	1:500	IF/Western blot
Epo	Proteintech; 17,908-1-AP	Rabbit	1:5,000	Western blot
Cleaved Cas-3	Merck; AB3623	Rabbit	1:100	Western blot
αTubulin	Sigma-Aldrich; T9026	Mouse	1:5,000	Western blot
Nanog	Cell Signaling, #D73G4	Rabbit	1:400	IF
OCT4A	Cell Signaling, #C53G3	Rabbit	1:1,600	IF
SMA	Sigma-Aldrich, #A2547	Mouse	1:100	IF
αFetoprotein	Dako, #A0008	Rabbit	1:100	IF
Neurofilament 200	Sigma-Aldrich; #N4142	Rabbit	1:400	IF
ß-III-tubulin/AF 594	Santa Cruz; #sc-80,005 AF594	Mouse	1:50	IF/FACS
Phantom dye red 780	Proteintech; #PD00002	/	1:1,000	FACS
Alexa Fluor 555	Thermo Fisher; #A32727	Mouse	1:1,000	IF
Alexa Fluor 594	Thermo Fisher; #A32732	Rabbit	1:1,000	IF
Alexa Fluor 633	Thermo Fisher; #A21070	Rabbit	1:1,000	IF
HRP	Sigma-Aldrich; #A4416	Mouse	1:10,000	Western blot

#### Transgenic iPSC characterization

To confirm pluripotency of the newly generated transgenic lines (namely CRLF3 KO and corresponding Ig Ctrl of iPSC#1 and #2) we stained for pluripotency markers NANOG and OCT4A (see antibody list in Table 3). iPSC were grown on 2 cm glass coverslips (Menzel-Gläser, #CB00200RA1) and fixed when confluent in 4% Paraformaldehyde (PFA) for 30 min. Coverslips were subsequently washed 3x in PBS before being blocked in 0.5 % bovine serum albumin (BSA; Thermo Fisher Scientific, #15260037) either for 30 min at RT or longer at 4°C. Cells were washed again 3 times in PBS and subsequently incubated with primary antibody according to Table 3 at 4°C overnight. Coverslips were washed 3 times in PBS before incubation with the corresponding secondary antibody (Table 3) at 37°C for 1 h. Cells were washed three times in PBS and once in water before mounting in Fluoromount-G (Thermo Fisher Scientific, #00-4,958-02). Images were taken with a Zeiss Observer Fluorescent microscope.

To show that differentiation capacities of transgenic lines remain intact after transgenesis we performed spontaneous differentiation assays, by embryoid body formation (EB), according to Rodriguez-Polo et al. (2019). In brief, iPSC colonies were detached with 200 U/mL Collagenase Type IV for 10 min at 37°C, scraped off and transferred to uncoated bacterial dishes. EBs were maintained in Iscoves medium (Thermo Fisher Scientific, # 12440053) at 37°C and the medium changed every second day.

After 8 days EB were transferred onto Geltrex-coated 6-well plates equipped with 2 cm coverslips for spontaneous differentiation and further maintained in Iscove’s Medium. Cells were fixed between day 18 and 20. Stainings were performed as described above. Spontaneously differentiated cells were stained for Smooth muscle actin (SMA) and α-Fetoprotein according to Table 3.

### Neuronal differentiation and survival-assay establishment

iPSC were differentiated as described previously (Qi et al., 2017) with slight modifications of the original protocol. iPSC were split on 12-well plates and maintained in UPPS until reaching confluency of 60%–80%. Medium was changed every 2nd to 3rd day. For the first 7 days of differentiation, cells were maintained in induction medium consisting of DMEM/F12 (Thermo Fisher Scientific; #11320033), 10% Knock out serum (KOS; Thermo Fisher Scientific; # 10828028), 1% Non-essential amino acids (NEAA, Thermo Fisher Scientific;# 11,140,050), 200 μM L-Ascorbic Acid (L-AA, Sigma-Aldrich; # A92902-100G), 2 μM SB431542 (Peprotech; # 3014193), 3 μM Chir99021 (Sigma-Aldrich, # SML1046) and 1.5 μM dorsomorphin (Peprotech, # 8666430). Cells were split onto fresh Geltrex-coated plates on day 6. Neuron splitting was performed following incubation in 0.25% Trypsin/EDTA (Thermo Fisher Scientific; # 25200056) for 3 min at 37°C. Cells were scraped and carefully resuspended before collection in a 5 mL falcon containing 5 ml DMEM/FBS. Cell suspension was centrifuged for 5 min at 200 × g. The supernatant was discarded, cells were resuspended in Induction medium +0.001 % ß-mercaptoethanol (Thermo Fisher Scientific; #21985023) and seeded onto 6-well plates. Medium was changed the next day to neuralization medium containing DMEM/F12, 200 μM L-AA, 1% NEAA, 1X N2 supplement (Thermo Fisher Scientific; # 17502048), 1X B27 supplement (Thermo Fisher Scientific; #17504044), 10 ng/mL bFGF (Peprotech; #100-18B) and 10 ng/ml EGF (Peprotech; #AF-100-15). Cells were fed with neuralization medium for 1 week before switching to neuronal differentiation medium I, consisting of DMEM/F12, 200 μM L-AA, 1% NEAA, 1X N2 supplement, 1X B27 supplement, 300 ng/mL cAMP (Peprotech; #6099240). For the final 7 days of neural differentiation, cells were maintained in neural differentiation medium II containing DMEM/F12, 200 μM L-AA, 1% NEAA, 1X N2 supplement, 1X B27 supplement, 300 ng/ml cAMP, 10 ng/ml BDNF (Peprotech; #450-02) and 10 ng/mL NT-3 (Peprotech; #AF450-03). During the differentiation process, cells were split once on Poly-L-Lysine/Laminin-coated plates when reaching 100% confluency. For each experiment 4 6-well plates were first coated in 1 μg/mL Poly-L-Lysine (Sigma-Aldrich; #P5899) for 30 min at 37°C. Subsequently, plates were washed 3x with PBS before being coated with 2 μg/ml Laminin (Sigma-Aldrich; #11243217001) for at least 8 h at RT in the dark. Before cells were seeded, plates were washed twice in PBS. Cell splitting was performed as described above. Differentiations were regularly monitored for differentiation progress using an inverted light microscope (Carl Zeiss; #4001648). Characterization of the emerging iPSC-derived neurons was performed by immunofluorescent stainings for ß-III-tubulin, Neurofilament and CRLF3 as described above (Table 3).

#### Establishment of survival assay

Different concentrations of rotenone as a pro-apoptotic stressor and EV-3 as an anti-apoptotic protectant were tested. For final experiments rotenone (Sigma-Aldrich; #R8875; dissolved in DMSO at stock concentration of 1.3 M) concentrations of 800 nM (for iPSC#1) and 1 μM (for iPSC#2) were applied for 18 h after treating cells with either 41.5 ng/mL (iPSC#1) or 33.3 ng/mL (iPSC#2) EV-3 (IBA GmbH, Göttingen, Germany) for 12 h. For each experiment one well of differentiations were treated with 0.006% DMSO as rotenone solvent control. After treatment periods, iPSC-derived neurons were prepared for FACS analysis as stated below.

For comparison with EV-3 treatments, recombinant human Epo (NeoRecormon; Roche) was applied in the same concentration as EV-3 to each iPSC-derived neuron line for 12 h before cells were stressed by addition of 800 nM or 1 μM rotenone.

#### FACS sample preparation and analysis

To collect samples for FACS analysis cell cultures were incubated in 0.25% Trypsin/EDTA for 3 min at 37°C before stopping the reaction with DMEM/FBS. Cells were scraped and resuspended by gentle pipetting before being transferred to falcon tubes. 2 mL DMEM/FBS were added and samples were centrifuged at 800 × g for 5 min. Samples were subsequently washed twice in PBS, with centrifugation steps between washing steps. In order to have samples for all treatment groups and to set FACS gates, only a subset of the cells were stained for further analysis. Samples designated for live/dead analysis were stained in Phantom dye Red 780 (Proteintech; #PD00002) for 30 min at 4°C according to the antibody list in Table 3. Samples were subsequently diluted with 2 ml PBS + 0.1 % FBS and centrifuged at 800 × g for 5 min. Samples were washed one more time in PBS/FBS before being blocked alongside with unstained samples for at least 1 h at 4°C in PBS/0.5% BSA. 5 mL PBS were added and samples were centrifuged before a second PBS washing step. Subsequently, samples stained for ß-III-tubulin as neuronal marker were incubated with antibody according to Table 3 overnight at 4°C. Samples that did not receive staining solution remained in blocking buffer. The next day 2 ml PBS were added to all falcons and the samples were centrifuged. After a second PBS washing step, cells were resuspended in FACS buffer (Containing PBS + 0.5% BSA + 2 mM EDTA) and strained through a 40 μm cell strainer (Sarstedt; #833945040) into FACS tubes (Fisher Scientific; #10579511). Samples were kept on ice until analysis with Sony cell sorter SH800S.

FACS gates were set according to the forward and sideward scatter measured in the main gate for single-cell analysis. Gates for the selection of ß-III-tubulin-positive cells were set according to the unstained control samples. Phantom dye gates for live and dead cells were set according to the unstressed population. For all samples 100,000 cells were measured. Only ß-III-tubulin positive cells (i.e., neuron-like cells) were analyzed for their survival according to Phantom dye staining.

FACS data is presented as boxplots showing the median cell survival, upper and lower quartile and whiskers representing 1.5 × interquartile ranges. Single data points are shown as circles within the boxplot. Cell survival data was normalized to the corresponding untreated control, which was set to 1.

#### CRLF3 immunostaining of iPSC-derived neurons

iPSC-derived neurons were grown on glass coverslips and fixed on day 30 of differentiation. Cell staining was performed as described above using primary antibodies for Neurofilament 200 and CRLF3 (see antibody list Table 3) and Dapi (1:1000 in H_2_O; Sigma Aldrich; #D9564) as nuclear marker. Images were taken using Leica SP8 confocal microscope (Leica Microsystems). Images were further processed using ImageJ.

#### Labeling of EV-3 and endocytosis assay

EV-3 was fluorescently labeled using the NanoTemper Monolith protein labeling Kit Red-NHS (NanoTemper; #MO-L011) according to the manufacturer’s instructions. Binding of cytokine and dye was performed over night at 4°C. The labeled cytokine was eluted from the monolith columns as instructed. The labeled protein was subsequently concentrated using molecular weight cut off filters (10 kDa cut off; Corning; #431477). Labeled EV-3 was aliquoted and stored at −80°C until further usage.

Endocytosis assays was performed as described previously in Miljus et al. (2017). In brief, iPSC-derived neurons that were split onto coated coverslips were treated with 10 mM EDTA for 15 min at 37°C on day 30. EDTA treatment was performed to inhibit calcium-dependent exocytosis. Subsequently, 8 μM FM1-43 (Biomol; #ABD-21483) was added directly into the medium together with 1 μM EV-3-647 conjugate. Cell cultures were incubated for another 10 min at 37°C, before briefly washing three times with PBS. Cultures were fixed subsequently and nuclei were labeled with Dapi as described above.

For all staining experiments one culture originating from the same differentiation remained untreated but received FM1-43 to ensure specificity of the EV-3/647 signal.

Stainings were imaged with a Leica SP8 confocal microscope (Leica Microsystems) and images were further processed using ImageJ.

### Gene expression studies

#### RNA isolation and cDNA synthesis

For each treatment group in the survival assays cells from two wells of a 6-well plate were pelleted for further molecular analysis. RNA was isolated by means of Trizole/Chloroform protocol as described previously (Knorr et al., 2021). In brief, 1 mL Trizole (Thermo Fisher Scientific; #15596026) was added to each cell pellet and cells were disrupted in a tissue lyser. 200 μL Chloroform (Labsolute; #2475) was added and the samples were shaken vigorously for 20 s in tissue lyser. Samples were incubated on ice for 15 min before centrifugation at 12,000 × g for 15 min at 4°C. The top translucent phase of each sample was transferred to a fresh Eppendorf cup and mixed with 1 mL ice-cold 75% EtOH. Samples were incubated at –20°C for at least 1 h before centrifugation at 10,000 × g for 15 min at 4°C. The resulting RNA pellet was washed three times in ice-cold EtOH before pellets were dried and resuspended in 30 μL ddH_2_O. RNA concentrations were measured by Nanodrop (Thermo Fisher Scientific).

cDNA was synthesized using the LunaScript RT SuperMixKit (New England BioLabs; #E3010) according to the manufactures instructions. For all samples 1 μg RNA was reverse transcribed.

#### qPCR

qPCR analyses of transcripts from *BAX, Caspase 3, BCL-2* and *CRLF3* were run using specific primers (see oligonucleotide list Table 1). The housekeeping gene (HKG) ß*-Actin* was used as reference. All primers were analyzed for their efficiencies previously. All samples were loaded in triplicates and (-) RT controls and water were run as negative controls on each plate. qPCRs reactions were prepared with final concentrations of 5 μL Luna® Universal qRT-PCR Master Mix (New England Bio-Lab; #M3003), 0.1 mM forward and reverse primers and 10 ng cDNA. qPCRs were pipetted in a 96-well clear well plates (StarLab; #E1403-5200) and run using a Bio-Rad CFX Connect Real-Time system (Bio-Rad; #1855201).

Ct values were analyzed using the Pfaffl method (Pfaffl, 2001) and data was normalized to the corresponding HKG value of each sample and further to the corresponding gene of interest (GOI) control value. Relative gene expression data is represented as Bar plots showing the geometric mean and standard deviations.

### Quantification and statistical analysis

Statistical analyses of all experiments conducted in this study was performed using R Studio (RStudio Team, 2015; R Core Team, 2019) and pairwise permutation test (two-tailed) within the packages coin and rcompanion (Zeileis et al., 2008; Mangiafico, 2019). In order to avoid false-positive results due to multiple comparisons, Benjamini Hochberg correction was included in all statistical calculations. Significant differences are shown by asterisks, with * indicating *p* < 0.05, ** *p* < 0.01 and *** *p* < 0.001. All data presented was collected from independent experiments. Only experiments with a minimum of 5 % survival loss in rotenone treated cultures were included into final analysis. Exact *n* values (defined as number of individually measured experiments) for each experiment can be found in the figure legends.

## Results

### Characterization of CRISPR-generated cell lines

**Figure 1:**
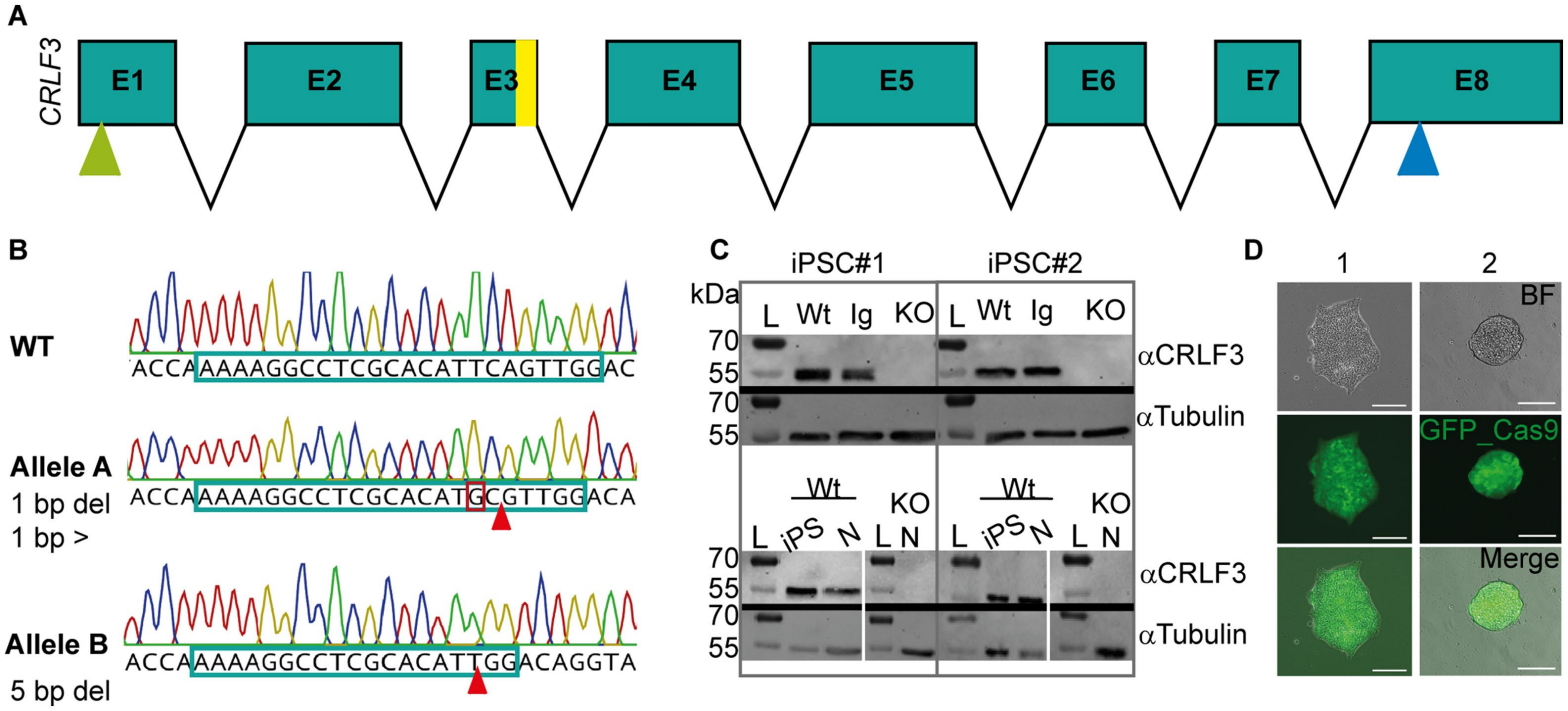
Characterization of CRISPR-induced CRLF3 mutation in human iPSC lines. **(A)** Schematic overview of the CRLF3 gene. Exons are represented as boxes, with sizes corresponding to exon length. Introns are represented as arrows and do not depict intron length. Yellow bar in exon 3 (E3) shows mutation site. Green and blue arrows mark start and stop codon of the coding sequence, respectively. **(B)** Chromatograms illustrating mutations in alleles A and B of iPSC#1 line. **Top**: WT sequence, with gDNA scaffold marked by framed portion of the nucleotide sequence. **Middle**: Allele A of the mutated line, which is characterized by one deleted base pair (marked with arrow) and a base pair exchange (marked by red box). **Bottom**: Allele B of the same iPSC line lacking a row of 5 deleted base pairs. Both mutations induce frameshifts that generate premature stop codons. **(C)** Western blot analysis of Ig-Ctrl and mutated iPSC lines and differentiated neurons. L, Ladder. **Top**: Protein lysates of iPSC#1 and #2 probed for CRLF3 and αTubulin. WT and Ig-Ctrl lysates show bands for CRLF3 protein, while the mutated lines (marked with KO) do not. αTubulin probed as loading control appears for all lines. **Bottom**: Western blot analysis of differentiated neurons originating from WT or CRLF3 mutated cells of both lines. While WT lysates show bands for CRLF3, no protein was detected in KO cells. αTubulin bands are present. **(D)** Transfected iPSC colonies express EGFP as reporter gene. Both lines show homogeneous eGFP expression within iPSC colonies consisting of more than 100 cells. Scale bar: 50 μM.

In order to generate CRLF3 deficient cell lines, two independent human iPSC lines (#1 and #2, female and male respectively) were transfected with a plasmid coding for the piggyBac transposase, and a piggyBac vector containing Cas9-GFP and gRNAs to target exon 3 of the gene (see Figure 1A). Figure 1 shows a representation of the mutation characterizations performed. Only cell lines showing two types of mutation (corresponding to alleles A and B) were considered for further analysis. Both generated KO lines contained frameshift-inducing mutations at the scaffold site resulting in termination of protein translation due to premature stop codons. Expression of CRLF3 protein in the generated cell lines was analyzed by Western blots. Both clonal KO lines generated from iPSC#1 and iPSC#2 entirely lack CRLF3 protein (Figure 1C). In contrast, CRLF3-related immunoreactivity of the expected size of 55 kDa was detected as single bands of WT and Ig-Ctrl cells. αTubulin was probed as loading control and detected at the expected size in all cell lines. The newly generated clonal lines all express GFP homogeneously, allowing to monitor cross-contaminations with other cell lines (see Figure 1D). Both Ig-Ctrl and KO cells from iPSC#1 and #2 were characterized for their pluripotent state (see Supplementary Figure 1). All four lines retained the capacity for spontaneous differentiation into all three germ layers (Supplementary Figure 1A) and exhibited staining for core pluripotency markers (Supplementary Figure 1B).

### EV-3 induces CRLF3-mediated protection of human iPSC-derived neurons

EpoR, but not ßcR, is expressed in iPSC-derived neurons used in this study (data not shown). Instead of Epo, which activates both classical EpoR and alternative tissue-protective receptors, we used the human natural Epo splice variant EV-3 for the main experiments. EV-3 is unable to activate classical EpoR, stimulates anti-apoptotic mechanisms in mammalian neurons (*via* alternative Epo receptors) and has been demonstrated to mediate protection of insect neurons *via* binding to CRLF3 (Bonnas et al., 2017; Hahn et al., 2017; Heinrich et al., 2017). Before starting with main experiments, we established protocols for apoptosis induction with rotenone and EV-3-mediated cell protection by testing different concentrations and exposure periods separately for both lines (Supplementary Figure 2 shows results of these experiments). Best combinations for apoptosis induction and neuroprotection differed between the two lines and led to the following protocols for subsequent survival assays: iPSC#1-derived neurons were exposed to 41.5 ng/mL EV-3 starting 12 h before exposure to 800 nM rotenone for 18 h. iPSC#2-derived neurons were exposed to 33.3 ng/mL EV-3 starting 12 h before exposure to 1 μM rotenone. In these preliminary experiments EV-3 protected neurons from both iPSC lines during rotenone-induced chemical hypoxia indicating that alternative Epo receptors activate the protective intracellular pathways.

**Figure 2:**
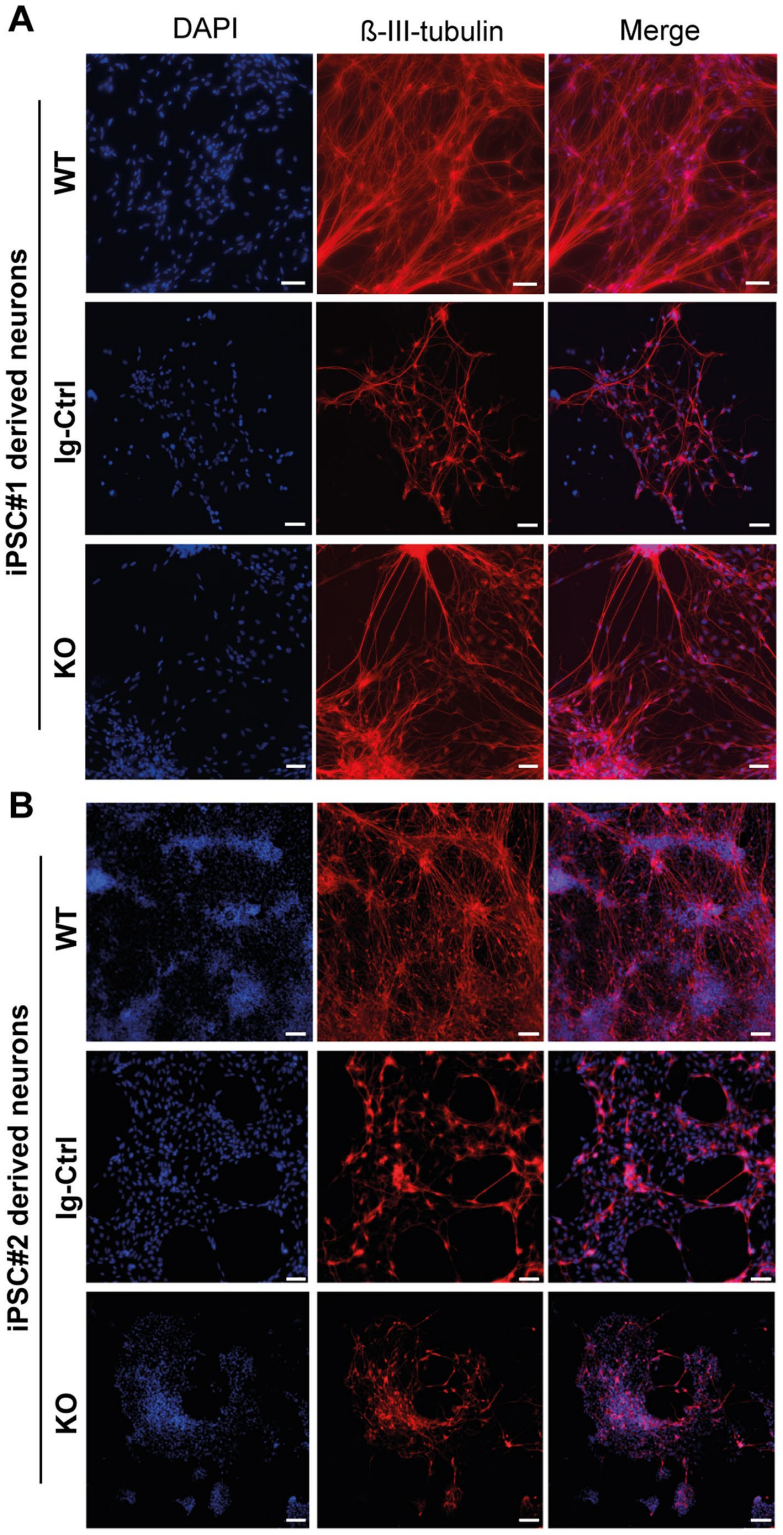
ß-III-tubulin stainings of iPSC-derived neurons. **(A)** Cells derived from iPSC#1. **(B)** iPSC#2-derived neurons. All generated cell lines contain extensive axonal networks. Scale bar 100 μM.

For core experiments WT, Ig-Ctrl and CRLF3 KO cells from both lines were differentiated for 30 days and subsequently treated with EV-3 according to our previous findings (41.5 ng/mL iPSC#1/33.3 ng/mL iPSC#2) EV-3. After 12 h 800 nM (iPSC#1) or 1 μM (iPSC#2) rotenone was added for 18 h before samples were collected for FACS analysis. 100,000 cells per sample were measured in five repetitions for each cell line. Of these, only ß-III-tubulin immunopositive cells were included in the quantitative analysis to circumvent variations resulting from divergent differentiation efficiencies (differentiation efficiencies are displayed in Supplementary Figure 3). Neuron-specific ß-III-tubulin staining of iPSC-derived neurons at day 30 of differentiation labeled extensive axonal networks of all lines (Figure 2).

**Figure 3:**
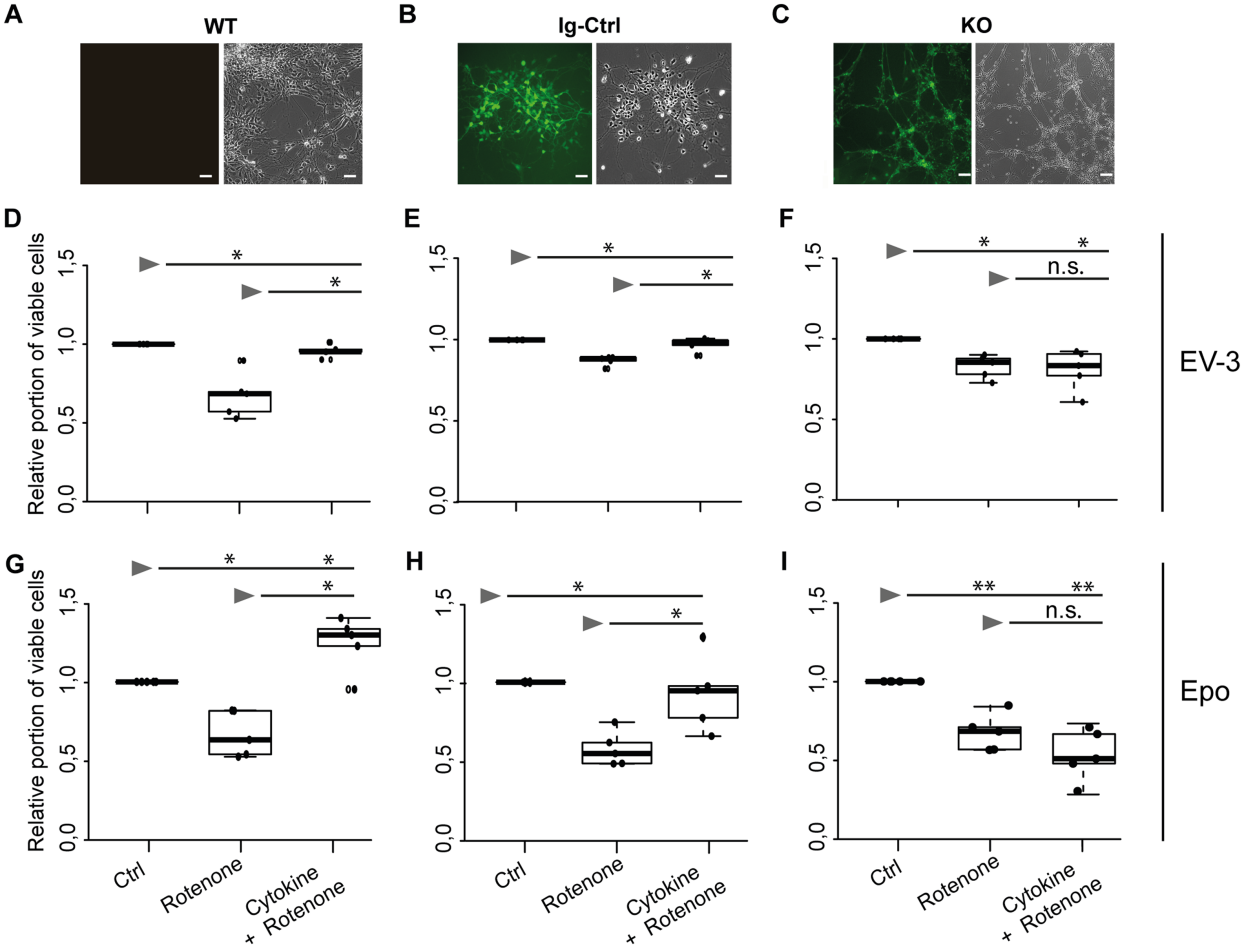
EV-3 and Epo induce CRLF3-mediated protection of human iPSC#1-derived neurons. Vertical panels depict data and images from WT **(A,D,G)**, isogenic controls (Ig-Ctrl) **(B,E,H)**, and CRLF3-mutated (KO) **(C,F,I)** iPSC-derived neurons. **(A–C)** Brightfield and fluorescent images of iPSC-derived neurons. Ig-Ctrl **(B)** and KO **(C)** cells display GFP fluorescence, indicating Cas9-GFP fuse transcript. **(D–I)** Relative survival of iPSC#1-derived neurons. Cells were prepared for FACS analysis by live/dead and ß-III-tubulin staining. **(D–F)** Data collected for neuron-like cells treated with EV-3. 18 h exposure to 800 nM rotenone reduced the survival of WT **(D)**, Ig-Ctrl **(E)**, and KO **(F)** neurons significantly when normalized and compared to survival in respective untreated control cultures. Rotenone-induced cell death was prevented by EV-3 (41.5 ng/mL) in WT and Ig-Ctrl neurons but not in CRLF3-KO neurons. *n* = 5 for all. **(G–I)** Relative survival of iPSC#1-derived neurons treated with Epo. 800 nM rotenone-induced cell death was prevented by 41.5 ng/mL Epo in WT **(G)** and Ig-Ctrl **(H)** cells. CRLF3-KO cells were not protected by Epo treatment. *n* = 5 for all. Data is presented as boxplots showing the median cell survival, upper and lower quartile and whiskers representing 1.5 × interquartile ranges. Single data points are shown as circles within the boxplot. Statistics with pairwise permutation test and Benjamini-Hochberg correction for multiple comparison. Significant differences: n.s, not significant, ^*^p < 0.05, ^**^p < 0.01.

Exposure to 800 nM rotenone reduced survival of WT, Ig-Ctrl and *CRLF3*-mutated iPSC#1-derived neurons (Figures 3D–F). WT neurons were particularly sensitive to rotenone treatment (median relative survival 0.68) compared to Ig-Ctrl (0.88) and KO (0.87) neurons. The deleterious effect of rotenone was completely prevented by EV-3 (41.5 ng/mL) in WT and Ig-Ctrl neurons (median relative survival 0.96 and 0.99). In contrast, EV-3 had no protective effect on *CRLF3* KO neurons (median relative survival 0.83) since cell survival was not different from rotenone-treated cultures (median relative survival 0.87).

Given that rotenone solutions are prepared in DMSO, control experiments with 0.006 % DMSO (represents the final concentration during treatments with 1 μM rotenone) had no impact on the survival of WT and Ig-Ctrl neurons compared to untreated cultures (median relative survival 0.99 and 1.02). Survival of *CRLF3* KO was slightly, yet significantly, decreased (median relative survival 0.96). However, the toxic effect of DMSO in these cells was not as severe as in rotenone exposed cultures (median relative survival 0.87). To study whether Epo also activates human CRLF3, we repeated the experiment with the same concentration as EV-3 in the previous experiment. Within this experimental series (Figures 3G–I) rotenone again reduced cell survival significantly in all three cell lines of iPSC#1 (Median cell survival: 0.67 WT; 0.65 Ig-Ctrl; 0.68 KO). Treatment with 41.5 ng/mL Epo 12 h before stressing rescued WT and Ig-Ctrl cells (1.24 and 0.94, respectively), while CRLF3-KO cells were not protected from undergoing rotenone-induced apoptosis (median cell survival 0.54).

**Figure 4:**
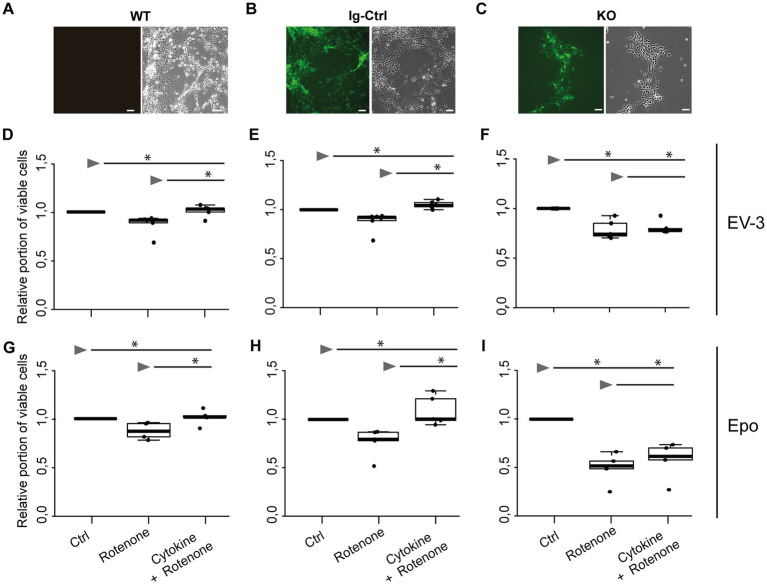
EV-3 and Epo induce CRLF3-mediated protection of human iPSC#2-derived neurons. Vertical panels depict data and images from WT **(A,D,G)**, isogenic controls (Ig-Ctrl) **(B,E,H)**, and CRLF3-mutated (KO) **(C,F,I)** iPSC-derived neurons. **(A–C)** Brightfield and fluorescent images of iPSC-derived neurons. Ig-Ctrl (B) and KO (C) cells display GFP fluorescence, indicating Cas9-GFP fuse transcript. **(D–I)** Relative survival of iPSC#2-derived neurons. Cells were prepared for FACS analysis by live/dead and ß-III-tubulin staining. **(D–F)** Data collected for neuron-like cells tested with EV-3. 18 h exposure to 1 μM rotenone reduced the survival of WT **(D)**, Ig-Ctrl **(E)**, and KO **(F)** neurons significantly when normalized and compared to survival in respective untreated control cultures. Rotenone-induced cell death was prevented by EV-3 (33.3 ng/mL) in WT and Ig-Ctrl neurons but not in CRLF3-KO neurons. Treatment of Ig-Ctrl neurons to EV-3 significantly increased cell survival in comparison to control cells. *n* = 5 for WT and KO experiments, *n* = 6 for Ig-Ctrl. **(G–I)** Relative survival of iPSC#2-derived neurons treated with Epo. 1 μM rotenone -induced cell death was prevented by 33.3 ng/ml Epo in WT **(G)** and Ig-Ctrl **(H)** cells. CRLF3-KO cells were not protected by Epo treatment. *n* = 5 for all. Data is presented as boxplots showing the median cell survival, upper and lower quartile and whiskers representing 1.5 x interquartile ranges. Single data points are shown as circles within the boxplot. Statistics with pairwise permutation test and Benjamini-Hochberg correction for multiple comparison. Significant differences: n.s, not significant, ^*^p < 0.05.

iPSC-derived neurons originating from iPSC#2 (Figures 4D–I) performed similarly in survival assays as cells originating from iPSC#1. Exposure to 1 μM rotenone, significantly decreased survival of WT, Ig-Ctrl and *CRLF3* KO neurons in comparison to untreated control cells (median survival 0.90, 0.92, 0.74, respectively). Treatment of WT and Ig-Ctrl cells with 33.3 ng/mL EV-3 rescued iPSC-derived neurons from rotenone-induced apoptosis, with cell survival of Ig-Ctrl cells being significantly increased to or beyond survival of untreated control cells (median survival 0.97 for WT and 1.04 for Ig-Ctrl cells). Treatment of *CRLF3* KO cells with EV-3 did not increase cell survival in comparison to sole rotenone exposure (0.78 median cell survival). Neurons derived from iPSC#2 reacted more strongly to DMSO treatment than iPSC#1 cells (see Supplementary Figure 4). Epo treatment also rescued WT and Ig-Ctrl iPSC#2-derived neurons (median survival WT rotenone 0.7 + Epo 1.03 Epo and Ig-Ctrl rotenone 0.75 + Epo 1.1). Knock out of CRLF3 prevented Epo-mediated protection of iPSC#2-derived neurons (rotenone ± Epo 0.60 and 0.67 median relative survival).

### EV-3 stimulates endocytosis in human iPSC-derived neurons

Stimulation of Epo-responsive cells has previously been reported to induce endocytosis of the ligand/receptor complex in both vertebrate and invertebrate neurons. In order to investigate if similar mechanisms are activated in EV-3 treated human iPSC-derived neurons, we labeled EV-3 with a 647 nm fluorophore, allowing us to visualize the location of the cytokine. iPSC-derived neurons were treated with labeled EV-3 together with the membrane dye FM1-43, which retains its fluorescence when the outer cell membrane is endocytosed.

**Figure 5:**
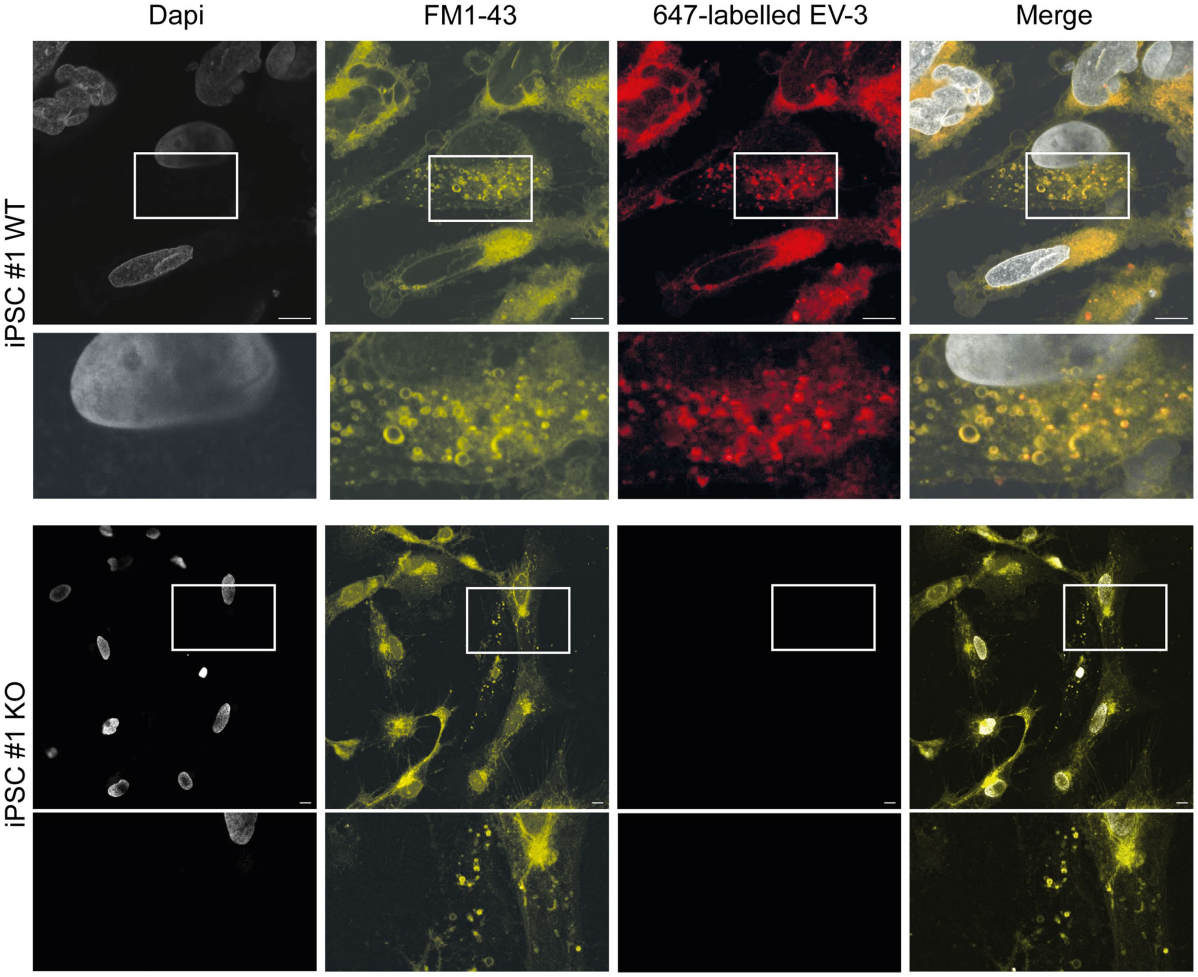
EV-3 induces endocytosis in iPSC-derived neurons. FM1-43 was used as membrane marker to visualize endocytosed vesicles. 647-labeled EV-3 was co-applied with membrane marker to visualize intracellular co-localization of EV-3 and vesicles. **Top row**: Exemplary image of iPSC#1 WT derived neurons, **Bottom row**: Neuron-like cells derived from iPSC#1 KO. Boxes depict enlarged areas displayed below the corresponding panels. Scale bars 10 μM.

Figure 5 depicts exemplary stainings of both WT human iPSC-derived neuron lines. Neuron-like cells incorporated FM1-43 stained vesicles, whose membranes were partially visible as fluorescent circles. EV-3-related fluorescence largely co-localized with the FM1-43 membrane marker, but appeared dot-like and more intensive inside the circular structures. CRLF3 KO cells did not accumulate endocytosed vesicles after treatment with EV-3. EV-3 fluorescence cannot be detected in KO cells (Figure 5).

KO cells show slightly altered morphologies (smaller somata and less extensive neural networks; visual observations), which have been observed in brightfield images as well (see Figure 3C and Figure 4C).

### CRLF3 protein levels in apoptogenic and rescue conditions

Potential treatment-related alterations of CRLF3 protein levels in WT and Ig-Ctrl iPSC-derived neurons were analyzed by Western blots. Samples from control and pharmacologically treated cultures of the same experiment were simultaneously analyzed on the same gel and blot. Both antibodies, anti-CRLF3 and anti-αTubulin for comparison, labeled single bands at the expected molecular size (~55 kDa for both proteins) in each sample.

**Figure 6:**
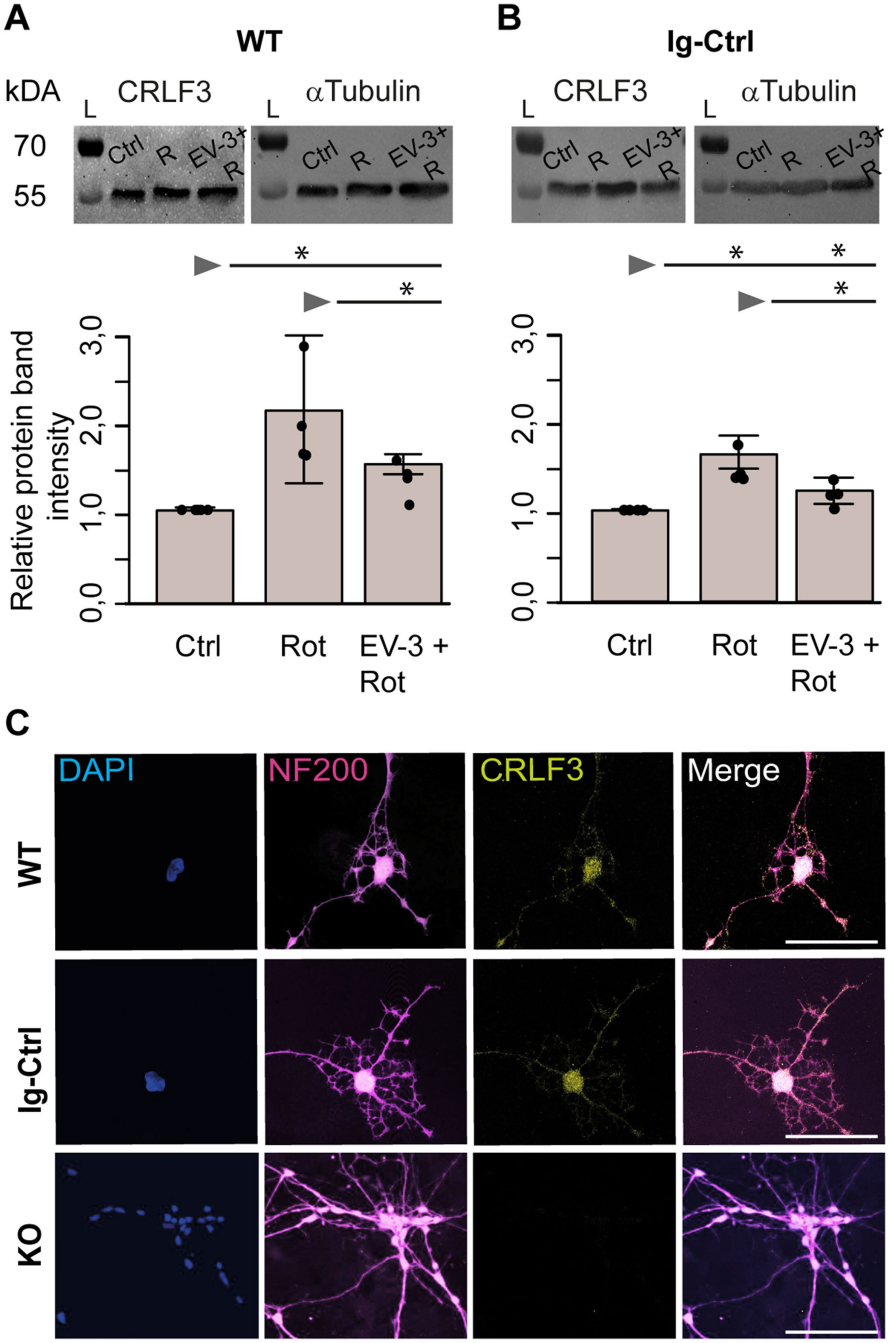
CRLF3 protein levels in apoptogenic and rescue conditions. Treatment with EV-3 and/or rotenone for 12 + 18 h started on day 30 of the differentiation protocol. Immediately after treatment, samples were collected. **(A,B)** Protein immunoblots of iPSC#1-derived WT **(A)** and Ig-Ctrl **(B)** neurons labeled with anti-CRLF3 (left) and anti-α-Tubulin (right) as loading control. Both antibodies labeled single bands of the expected molecular size (both ~55 kDa) in each sample. Rotenone (800 nM) increased CRLF3 protein levels in WT and Ig-Ctrl neurons. Co-treatment with EV-3 (41.5 ng/mL) reduced rotenone-induced CRLF3 accumulation insignificantly in WT and significantly in Ig-Ctrl neurons. *n* = 4. Data is shown as bar plots representing the average band intensities together with the calculated standard deviation and single data points. Statistics: pairwise permutation test with Benjamini-Hochberg correction for multiple comparison. Significant differences: ^*^p < 0.05. **(C)** Immunofluorescent labeling of neurofilament 200 (NF200; neuronal/axonal marker) and CRLF3 in all iPSC#2-derived neurons. Nuclei were labeled with Dapi. CRLF3 immunoreactivity in WT and Ig-Ctrl covers entire neurons with extensive labeling in the soma. No CRLF3 immunoreactivity is detected in KO cells. Scale bars 100 μM.

Exposure to rotenone increased CRLF3 levels in iPSC#1 WT (2.1 ± 0.7 fold) and Ig-Ctrl neurons (1.6 ± 0.2 fold) compared to untreated controls (Figures 6A,B). Rotenone-induced accumulation of CRLF3 was reduced by co-treatment with EV-3 in WT (1.5 ± 0.1 STDV, not significant compared to rotenone-only treatment) and Ig-Ctrl neurons (1.2 ± 0.1 STDV, significantly different from rotenone-only exposure). Given the comparatively low differentiation efficiency of iPSC#2, these experiments were only performed with iPSC#1-derived cells, to assure a sufficiently high proportion of neuron-like cells in analyzed samples.

In order to determine the localization of CRLF3 iPSC#1-derived neurons, we labeled differentiated WT, Ig-Ctrl and *CRLF3* KO cells with Dapi and antibodies against neurofilament 200 and CRLF3. As shown in Figure 6C, all cell lines expressed neurofilament 200, which is generated in the cell body cytosol and transported into axons. CRLF3-immunoreactivity appeared in dot-like patterns in cell bodies and along axons of WT and Ig-Ctrl cells but not in *CRLF3* KO cells. CRLF3 KO cells display slightly smaller somata and less dendrites (visual observations) compared to WT and Ig-Ctrl cells.

### Effects of rotenone and rotenone/EV-3-exposure on gene expression

We analyzed the expression of pro-and anti-apoptotic genes in untreated, rotenone-exposed and rotenone plus EV-3-treated iPSC-derived neurons. Analysis included early pro-apoptotic *BAX*, late pro-apoptotic *Caspase 3*, anti-apoptotic *BCL-2* and *CRLF3*. Figure 6 displays the data as average relative gene expression with standard deviation. Given the comparatively low differentiation efficiency of iPSC#2, these experiments were only performed with iPSC#1-derived cells, to assure a sufficiently high proportion of neuron-like cells in analyzed samples.

**Figure 7:**
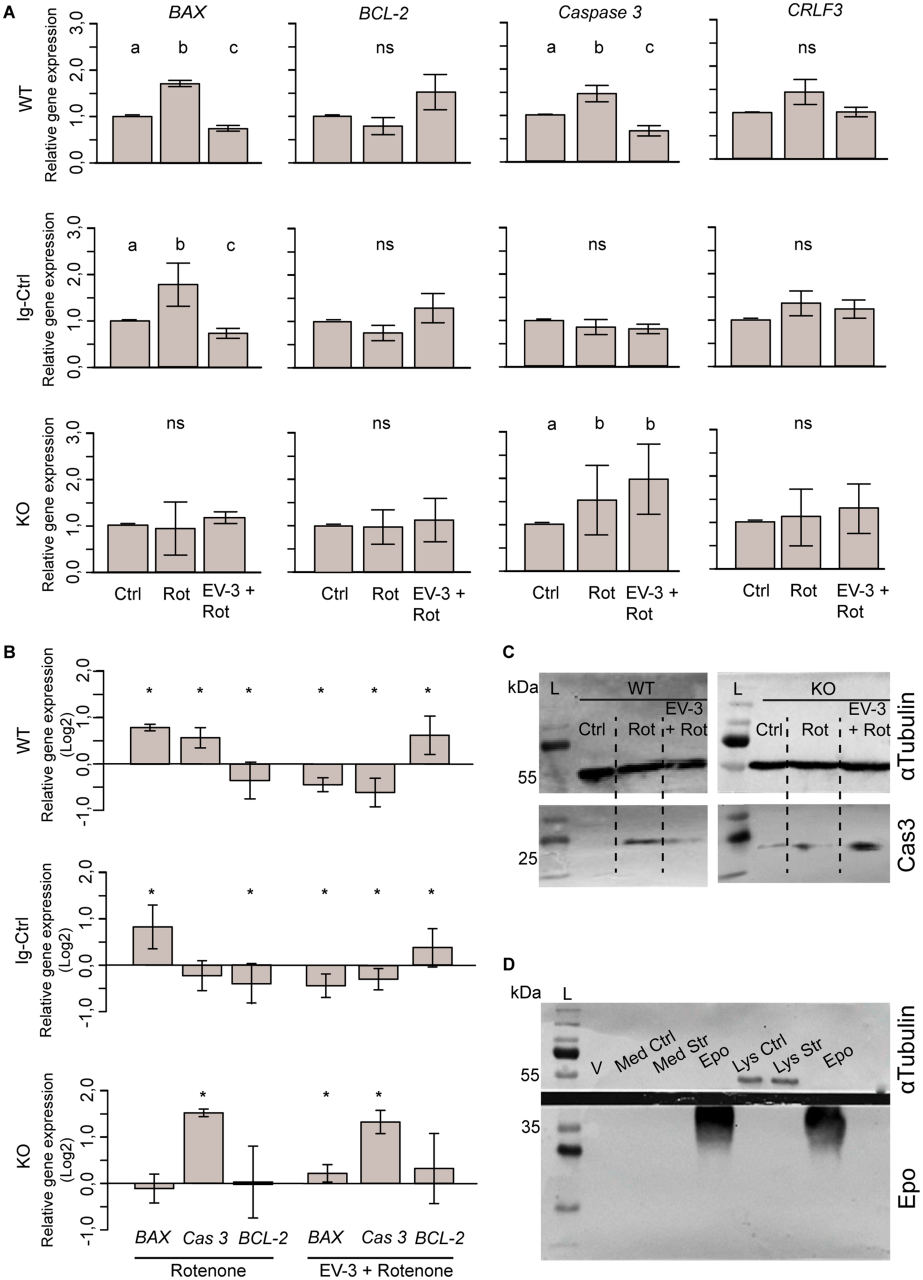
Expression of pro-and anti-apoptotic genes and *CRLF3* in iPSC#1-derived neurons. **(A)** qPCR-based relative gene expression of pro-apoptotic *BAX* and *Caspase 3*, anti-apoptotic *BCL-2* and *CRLF3* in WT, Ig-Ctrl and CRLF3 KO lines after exposure to rotenone ± EV-3 (12 h EV-3 treatment, followed by 18 h rotenone+EV-3 exposure). Values were normalized to untreated controls. n = 4 for each cell line. Graphics show average ± STDV; statistics with pairwise permutation test and Benjamini-Hochberg correction for multiple comparison. Significant differences (p < 0.05) are indicated by differing lettering. **(B)** Same data as in **(A)** illustrated as log2 for direct comparison of GOI up-and down regulation between different treatments of WT, Ig-Ctrl and KO neurons. Relative gene expression in pharmacologically treated cultures were only compared to respective untreated controls. Graphics show average ± STDV; Statistics with *t*-test. Significant differences (*p* < 0.05) are indicated by asterisk. **(C)** Exemplary Western blot analysis of cleaved caspase 3 FIGURE 7 (Continued)using iPSC#1-derived WT and KO neurons stressed with rotenone (Rot) or co-treated with EV-3 (EV-3 + Rot). While WT control lysates (Ctrl) lack signs of activated caspase 3 (Cas3), lysates from purely rotenone-and rotenone+EV-3-treated cells contain activated caspase 3-like immunoreactivity at the expected size of ~20 kDa (bottom membrane). KO cells show protein bands for active caspase 3 in all samples, with EV-3 treated neurons displaying the most intense band. All lysates show α-Tubulin bands at around ~55 kDa. 100 μg protein was loaded to all lanes. **(D)** Anti-Epo probed Western blot analysis of virgin medium (V), medium of control and rotenone-stressed cells (Med Ctrl, Med Str) and lysates of control and rotenone-stressed cells (Lys Ctrl and Lys Str) lack bands of Epo-like immunoreactivity. Solutions of rhEpo generated immunoreactive bands at ~40 kDa. 100 μg protein was loaded for all samples.

Expression of each gene of interest is separately displayed in Figure 7A. Expression of pro-apoptotic *BAX* significantly increased during exposure to the apoptogenic rotenone stimulus in WT (1.7 fold ±0.06 STDV) and Ig-Ctrl neurons (1.76 fold ±0.46 STDV) compared to respective untreated cultures. Co-application of EV-3 prevented rotenone-induced *BAX* overexpression and caused a significant decrease of relative expression compared to untreated controls (WT 0.74 ± 0.06, Ig-Ctrl 0.74 ± 0.12 STDV). In contrast, *BAX* expression in CRLF3-deficient KO neurons was neither altered by rotenone (0.93 ± 0.56 STDV) nor by combined treatment with rotenone and EV-3 (1.16 ± 0.13 STDV). Expression of executioner caspase *Caspase 3* in WT cells significantly increased during rotenone exposure (1.47 ± 0.17 STDV) and was reduced below control level following exposure of rotenone with EV-3 (0.66 ± 0.11 STDV). Neither of the treatments altered *Caspase 3* expression in Ig-Ctrl cells. *CRLF3*-KO cells significantly increased *Caspase 3* expression during rotenone exposure (1.52 ± 0.74 STDV), with overexpression not being prevented by co-treatment with EV-3 (1.97 ± 0.75 STDV). *BCL-2* and *CRLF3* expression were not significantly altered by exposure to rotenone or rotenone plus EV-3 in WT, Ig-Ctrl and CRLF3 KO iPSC-derived neurons. However, *BCL-2* expression in rotenone/EV-3-treated WT (1.52 ± 0.18 STDV) and Ig-Ctrl cells (1.3 ± 0.32 STDV) seemed to be elevated, especially compared to rotenone-only treated cultures. WT and Ig-Ctrl lines also demonstrate slightly elevated CRLF3 expression (not significant) upon exposure to the rotenone stressor (1.46 ± 0.27 and 1.39 ± 0.27 STDV respectively).

For direct comparison of treatment-related changes of gene expression, we rearranged the data in Figure 7B and plotted it as the log2 of relative gene expression compared to untreated controls. While WT cells significantly increase the expression of both early and late apoptosis genes when stressed (*BAX* 0.77 ± 0.07; *Caspase 3* 0.55 ± 0.22), Ig-Ctrl and KO cells overexpress either *BAX* or *Caspase 3, respectively.* Anti-apoptotic *BCL-2* expression was downregulated in WT and Ig-Ctrl cells and unchanged in KO cells. When rotenone-exposed cells are treated with EV-3, the overexpression of pro-apoptotic genes is prevented and anti-apoptotic *BCL-2* is significantly overexpressed in WT and Ig-Ctrl cells (0.6 ± 0.4 and 0.38 ± 0.4 STDV respectively). *CRLF3-*KO cells overexpress *Caspase 3* when treated with rotenone+EV-3 (1.29 ± 0.25 STDV). *BCL-2* and *BAX* expression remain elevated in comparison to control (0.31 ± 0.74 and 0.22 ± 0.18 STDV, respectively).

Given that caspase 3 is subject to post-translational modifications mediating its activation for execution of apoptotic cell death, we probed the presence of active (cleaved-) caspase 3 in cell lysates of iPSC#1-derived neurons. A clear band for active caspase 3 can be seen for rotenone-treated WT neurons, while Ctrl cells show no reactivity to active caspase 3 (Figure 7C). EV-3 treated cells only show a very indistinct band for active caspase 3. KO neurons show immunoreactivity to cleaved caspase 3 in all lysated, with cells treated with EV-3 additionally to rotenone show the highest band intensity. All lysates contain α-Tubulin bands at the expected size of ~55 kDa.

To ensure that our experiments were not affected by production and release of Epo from the cultured neuron-like cells, we collected cell culture medium and lysates of control and rotenone-stressed cells. After precipitation 100 μg protein from each sample were loaded to the gel. Epo was loaded at the same concentration as control. As expected, only cell lysates showed α-Tubulin bands (top membrane, ~55 kDa). No Epo-positive band could be detected except for lanes that were loaded with Epo solution (Figure 7D; ~40 kDa).

## Discussion

The cytokine Epo mediates neuroprotection and promotes regeneration in mammalian nervous systems. Animal models and clinical observations identified Epo as a promising treatment option to prevent neurodegenerative cell loss. While systemically administered Epo co-activates adverse effects such as overproduction of blood cells increasing the risk of thrombosis and promotion of tumor growth, some Epo-mimetics including EV-3 selectively stimulate tissue protection without activating homodimeric EpoR-associated side effects. Hence, identification and selective targeting of tissue-protective Epo receptors should be attempted for therapies against neurodegenerative diseases. The present study identifies EV-3/CRLF3-signaling in human neurons as a promising neuroprotective option.

### CRLF3 as a conserved neuroprotective cytokine receptor

Previous studies on insects suggested that the evolutionary conserved orphan cytokine receptor CRLF3 may serve as neuroprotective receptor for Epo in the mammalian nervous system (Hahn et al., 2017, 2019). CRLF3 has been associated with a variety of diseases including neurofibromatosis type I, cutaneous Leishmaniasis, cutaneous squamous cell carcinoma, amyotrophic lateral sclerosis, autism and cancer (Dang et al., 2006; Serra et al., 2019; Castellucci et al., 2021; Kehrer-Sawatzki et al., 2021; Wegscheid et al., 2021). Apart from this, studies on PC12 cells and iPSC-derived cerebral organoids indicated that CRLF3 regulates the development and differentiation of neurons (Wegscheid et al., 2021). However, concrete functions of CRLF3 remained unknown until its regulatory role in vertebrate hematopoiesis and particularly in mammalian thrombopoiesis were recently reported (Bennett et al., 2022; Taznin et al., 2022), though without discovering the identity of its ligand. Insect CRLF3 initiates anti-apoptotic neuroprotective mechanisms upon activation with both human Epo and EV-3 (Hahn et al., 2017, 2019). While the endogenous ligand for insect CRLF3 is still unknown (Knorr et al., 2021) current knowledge suggests that CRLF3 is the only Epo/EV-3-responsive receptor in insects. In contrast, mammals express classical homodimeric EpoR activated by Epo and alternative tissue-protective Epo receptors activated by Epo and selective ligands such as EV-3. EV-3 is a natural Epo splice variant that lacks the entire third exon of the *Epo* transcript which prevents activation of homodimeric EpoR and heteromeric EpoR/ßcR (Bonnas, 2009; Bonnas et al., 2017). EV-3 is present in human serum and brain and elicits anti-apoptotic effects in rat hippocampal neurons (Bonnas et al., 2017). Using EV-3 in our study prevented the activation of EpoR which is expressed in both iPSC lines and iPSC-derived neurons, and EpoR/ßcR heteroreceptors, though ßcR expression was only detected in undifferentiated iPSC (data not shown). No evidence for potential production and release of Epo by iPSC-derived neurons was found by Western blot analysis of culture medium and cell lysates from normal and rotenone-stressed cell cultures. Hence, the observed effects of EV-3 were neither directly nor indirectly mediated through homodimeric or heterodimeric EpoR. In contrast, protective effects of Epo could be mediated by both CRLF3 and EpoR. Demonstrating that EV-3 mediates neuroprotection *via* human CRLF3 not only deorphanizes CRLF3 but also identifies the previously proposed neuroprotective receptor for EV-3 (given that both receptor and ligand are endogenously present in humans). Since Epo can be regarded as the more general ligand that stimulates both erythropoiesis and tissue protection and insect CRLF3 is activated by both Epo and EV-3, it was assumed that human CRLF3 will also be stimulated by EV-3 and Epo.

### Epo and EV-3 as neuroprotective CRLF3 ligands

EV-3 protects insect and rat neurons at similar or lower concentrations than Epo (Miljus et al., 2014; Bonnas et al., 2017; Hahn et al., 2017; Heinrich et al., 2017; Miljus et al., 2017). Both Epo and EV-3 protect neurons in an optimum-type dose response, with both lower and higher concentrations being less neuroprotective and very high concentrations even exerting deleterious effects on cell survival (Siren et al., 2001; Chong et al., 2003; Weishaupt et al., 2004; Bonnas et al., 2017; Hahn et al., 2017; Heinrich et al., 2017). Optimal concentrations may vary between species (e.g., brain neurons of *L. migratoria* and *T. castaneum*) and even between different cell types within the same organism and tissue (brain neurons and glia of *L. migratoria*). Such differences were also detected between the two lines of iPSC-derived neurons used in our study. While rotenone-stressed neurons of iPSC#1 were best protected by 41.5 ng/mL EV-3, the most neuroprotective concentration for iPSC#2 was 33.3 ng/mL. Apoptosis-induction with rotenone has frequently been used in studies with various cell types including neurons and Epo-mediated neuroprotection of rotenone-stressed neurons has been reported *in vitro* (Wen et al., 2002; Cheng et al., 2020). In this study, rotenone was chosen as a stressor to induce chemical hypoxia and to connect with previous studies that applied hypoxia directly (Miljus et al., 2014; Hahn et al., 2017, 2019). Rotenone is routinely applied in studies on mechanisms underlying Parkinson’s disease in order to explore the potential of neuroprotective compounds. Rotenone induces “chemical hypoxia” through inhibition of complex I of the mitochondrial respiratory chain which interferes with ATP production and generates reactive oxygen species. It is important to note, that iPSC#2 cells differentiated not as efficiently as cells originating from iPSC#1. This could account for the higher concentration of rotenone required, in order to sufficiently stress the cells for a significant portion of apoptosis induction. iPSC and differentiated cells originating from them are prone to highly heterogeneous differentiation efficiencies as well as physiological properties (Hayashi et al., 2019). This characteristic has been widely accepted within the scientific community. It was therefore not surprising that the two iPSC lines used in this study differentiated into neurons with different efficiency and required differing concentrations of both rotenone and EV-3 for apoptosis-induction and neuroprotection, respectively. Furthermore, it cannot be excluded that the sex difference between the lines (iPSC#1 being derived from a female donor and iPSC#2 originating from a male donor) caused some physiological differences between the respective neuron-like cells.

To our knowledge, the neuroprotective role of Epo or EV-3 in human neurons has not been directly studied. Aiming to explore the potential of Epo-mediated cytoprotection in human cells, we generated iPSC-derived neurons that recapitulate essential aspects of *in vivo* human neurons. Both lines of iPSC-derived neurons assumed neuron-like morphologies and expressed neuron-specific proteins including ß-III-tubulin and neurofilament 200. We strived to understand if (1) EV-3 elicits neuroprotective functions in human neuron-like cells and (2) if this neuroprotection requires the presence of human CRLF3. We demonstrate that EV-3 administration 12 h before and during rotenone-exposure protects WT and Ig-Ctrl neuron-like cells from stress-induced apoptosis. For both cell lines used in this study the apoptotic effect of rotenone was entirely compensated, resulting in cell survival close to control (untreated cells) levels. Importantly, EV-3-mediated neuroprotection was completely absent in CRLF3 KO cells. This data provides evidence that EV-3 and CRLF3 represent a ligand-receptor pair that stimulates protective mechanisms in human cells. Given that Epo has been reported to induce neuroprotection in insect neurons *via* CRLF3 (Hahn et al., 2017, 2019), we additionally tested whether CRLF3 was also involved in human Epo-mediated neuroprotection. Our data demonstrates that, similar to EV-3 treated neurons, Epo was able to rescue WT and Ig-Ctrl cells from both iPSC-derived neuron lines from rotenone-induced stress. KO cells on the other hand were not protected, indicating the involvement of CRLF3 also in human Epo-mediated neuroprotection. Even though human iPSC-derived neurons express classical EpoR, its presence was insufficient to mediate significant Epo-stimulated neuroprotection in the absence of CRLF3. To the present, Epo-mediated neuroprotection of mammalian cells has been reported to be dependent on the activation of homodimeric EpoR and/or heterodimeric EpoR/ßcR (Brines et al., 2004; Nadam et al., 2007; Sanchez et al., 2009a,b; Chamorro et al., 2013; Miller et al., 2015; Ding et al., 2017). This study extends the repertoire of Epo-targeted neuroprotective receptors to CRLF3.

Since EV-3-mediated neuroprotection shares characteristics with Epo-mediated neuroprotection, we further studied if the presence of EV-3 in cell culture media induced endocytosis. Endocytosis of the Epo/EpoR complex has been described for mammalian hematopoietic cells (Sawyer et al., 1987). Similarly, experiments with locust neurons demonstrated that both human Epo and EV-3 stimulate endocytosis after binding to the same receptor in insect neurons (Miljus et al., 2017) that was later identified as CRLF3. To study potential ligand-induced receptor endocytosis in human neuron-like cells, we performed endocytosis assays with labeled EV-3/647. While CRLF3 KO cells did not show any endocytosed vesicles associated with EV-3 fluorescence after treatment, WT and Ig-Ctrl cells accumulated EV-3-associated fluorescent particles, that co-localized with FM1-43 labeled vesicle membranes. Although we did not directly confirm a physical interaction of EV-3 and CRLF3 this indicates that EV-3 initiates endocytosis upon binding to a cell membrane-located receptor. Since accumulation of EV-3-associated fluorescence was absent in CRLF3-KO cells, binding of EV-3 to CRLF3 is highly likely. It is to be noted that CRLF3 KO cells showed altered morphologies in comparison to WT and Ig-Ctrl cells (visual observations). This characteristic was observed not only in fluorescent stainings (as in Figures 5, 6) but also in bright field images of these cells (see Figures 3, 4). Given that CRLF3 has been associated with neuronal development and differentiation in earlier studies (Hashimoto et al., 2012; Wegscheid et al., 2021) it is likely that the changes in cell morphology are based in the lack of CRLF3.

Physiological and/or pathological stress elevates *EpoR* expression in neuronal cell cultures (Chin et al., 2000; Chen et al., 2010; Merelli et al., 2019), in spinal cord (Cohrs et al., 2018) and brain (Sanchez et al., 2009a; Merelli et al., 2019; Wakhloo et al., 2020). Additionally, increased presence of EpoR/ßcR in renal cells after ischemic reperfusion injury were also reported (Yang et al., 2013). Cell-protective Epo receptors were either upregulated (Xu et al., 2009) or downregulated (Yang et al., 2013) by the presence of Epo or receptor-activating Epo mimetic molecules in some studies. Hence, we asked whether *CRLF3* expression in iPSC-derived neurons was similarly affected by rotenone-induced stress and EV-3 application. Western blot analysis of iPSC#1 WT and Ig-Ctrl neurons indicated increased CRLF3 levels following rotenone-exposure and partial prevention of this increase by co-application of EV-3. In contrast to the survival assays that selectively analyzed neuron-like cells, Western blot analysis non-selectively included all cells in these cultures. Nevertheless, the data suggest that CRLF3 is upregulated under apoptogenic conditions. The presence of EV-3 reduced apoptosis induction by rotenone causing no or reduced upregulation of CRLF3 protein. However, no rotenone and/or EV-3 effects on CRLF3 levels were detected in iPSC#2 WT and Ig-Ctrl neurons. This result is probably caused by lower differentiation efficiency of iPSC#2 (compared to iPSC#1) and more diluted effects by higher portions of non-neuronal cell types.

### Basic characterization of CRLF3-mediated anti-apoptotic mechanisms

For an initial characterization of EV-3/CRLF3-mediated anti-apoptotic mechanisms we analyzed the expression of pro-and anti-apoptotic gene transcripts and proteins in neuron-like cells derived from iPSC#1. Initiation and progress of apoptosis has previously been correlated with altered gene expression of *Caspase 3* and *BAX* (Persad et al., 2004; Naseri et al., 2015; He et al., 2018; Hu et al., 2021). Our data suggest that 18 h rotenone-induced stress leads to overexpression of both “early” and “late” pro-apoptotic genes (namely *BAX* and *Caspase 3*) in WT cells. It is intriguing that in contrast to iPSC#1 WT cells, iPSC#1 Ig-Ctrl neuron-like cells seem to initiate apoptosis slower, which is underlined by the lack of *Caspase 3* overexpression but a pronounced overexpression of the early apoptosis marker *BAX*. For both WT and Ig-Ctrl cells, EV-3 prevented rotenone-induced elevation of *Caspase 3* and/or *BAX.* Additionally, anti-apoptotic *BCL-2* expression increased in cells treated with EV-3. Caspase 3 is present as an inactive proenzyme in physiologically intact cells. Proteolytic cleavage by initiator caspases activates its activity to execute apoptosis. Immunoblots against activated cleaved caspase 3 confirmed its absence in normally cultured iPSC-derived neurons and its emergence following rotenone exposure. EV-3 treated cells demonstrated a faint band for cleaved caspase-3. Even though transcriptional changes in pro-and anti-apoptotic genes have been studied and observed here, it cannot be excluded that EV-3, similar to Epo, also activates antioxidative pathways (e.g., upregulation of glutathione peroxidase; Thompson et al., 2020; Rey et al., 2021) which lead to the observed protection from rotenone-induced apoptosis. Given that earlier studies in insect neurons revealed involvement of JAK/STAT signaling in Epo and EV-3-mediated neuroprotection (Miljus et al., 2014, 2017), it is likely that human CRLF3 activates similar pathways.

This data suggests that EV-3 mediates neuroprotection by upregulation of anti-and downregulation of pro-apoptotic genes, enabling the cells to counteract apoptotic processes induced by rotenone. Interestingly, the iPSC#1 KO cells analyzed in this study do not display these protective gene expression profiles. *CRLF3*-KO cells show a rather dysregulated transcriptional program, indicated by a high variance among the different samples. Rotenone treatment did not result in overexpression of *BAX* but in a clear overexpression of *Caspase 3*. Neither *BAX* nor anti-apoptotic *BCL-2* were differentially expressed when KO cells were treated with EV-3 and *Caspase 3* remained strongly overexpressed. Immunoblots for active caspase 3 further verified our gene expression data and revealed cleaved caspase 3 protein in all experimental conditions. EV-3 and rotenone treated cells displaying the strongest caspase 3 bands. The lack of *BCL-2* overexpression in EV-3 treated cells together with the high variances observed in gene expression profiles not only underline the absence of any EV-3 mediated cell-protective effects but also points toward regulatory functions of CRLF3 in cell homeostasis.

The data presented here identify human CRLF3 as a receptor for the natural Epo splice variant EV-3. Expression of CRLF3 in various human tissues suggests that CRLF3-stimulated transduction pathways can interfere with apoptotic processes in other cell types besides neurons.

The involvement of CRLF3 in human iPSC-derived neuroprotection will initiate a variety of studies to uncover the protective molecular pathways. Epo-mediated protection of mammalian neurons reported in numerous previous studies may have been mediated by EpoR and/or EpoR/ßcR and/or CRLF3 unless control experiments associated the observed neuroprotective effects with the activation of a particular type of Epo-responsive receptor. Implication of CRLF3 in anti-apoptotic and cell-protective mechanisms will facilitate the identification of additional Epo-like ligands to be applied as specific neuro-or other tissue-protective agents. Using iPSC-derived cell types from healthy and diseased donors enables focused studies on cell-protective mechanisms in cell-specific molecular settings.

## Data availability statement

The original contributions presented in the study are included in the article/Supplementary material, further inquiries can be directed to the corresponding authors.

## Author contributions

DK, IRP, RB, and RH designed and supervised the study. DK, IP, HP, SP, and NS-D performed experiments. DK, IRP, and HP analyzed the data. DK, IRP, and RH wrote the manuscript. All authors contributed to the article and approved the submitted version.

## Funding

This work was supported by the Deutsche Forschungsgemeinschaft (DFG; project numbers: 398214842 and 499371712).

## Conflict of interest

The authors declare that the research was conducted in the absence of any commercial or financial relationships that could be construed as a potential conflict of interest.

## Publisher’s note

All claims expressed in this article are solely those of the authors and do not necessarily represent those of their affiliated organizations, or those of the publisher, the editors and the reviewers. Any product that may be evaluated in this article, or claim that may be made by its manufacturer, is not guaranteed or endorsed by the publisher.

## Supplementary material

SUPPLEMENTARY FIGURE S1 Characterization of newly generated iPSC lines. For both line 1 and 2, Ig-Ctrl and *CRLF3* KO cells were examined to ensure full pluripotency. **(A,B)** iPSC of all four lines were stained for two characteristic pluripotency markers. All cells show clear nuclear staining for Nanog and OCT4A. **(C,D)** Ig-Ctrl and KO cells of both lines were used to form embryoid bodies and allow spontaneous differentiation. Cells were stained for characteristic markers of the three germ layers. All four lines generate immunopositive cells for alpha-fetoprotein (AFP) representing endodermal tissue and smooth muscle actin (SMA) representing mesoderm. ß-III-tubulin stainings for ectodermal differentiation are presented in Figure 2 of the main manuscript. Scale bars: 20 µM.

SUPPLEMENTARY FIGURE S2 Survival assays with iPSC-derived neurons. iPSC lines 1 and 2 (left and right column respectively) were differentiated into neurons and subsequently (on day 30 of differentiation) treated with either rotenone alone or rotenone and EV-3. **(A1,A2)** iPSC-derived neurons were treated with either 800 nM or 1 µM rotenone for 18 h. Cell survival was measured using FACS. Survival in both cell lines is significantly reduced when cells receive 800 nM rotenone. 1 µM rotenone significantly decreases cell survival in line 2, however not in line 1. *n* = 5 for both lines. **(B1)** Cell death induced by 800 nM rotenone is unaffected by 33.3 ng/ml EV-3 but completely prevented by 41.5 ng/ml EV-3 in iPSC-derived neurons of line 1 (*n* = 4). **(B2)** Cell death induced by 800 nM rotenone is partially prevented by both concentrations of EV-3 in line 2 iPSC-derived neurons (*n* = 8). **(C1)** Cell death induced by 1 µM rotenone is unaffected by EV-3 in line 1 iPSC-derived neurons (*n* = 4). **(C2)** Cell death induced by 1 µM rotenone is unaffected by 41.5 ng/ml EV-3 but partially reduced by 33.3 ng/ml EV-3 (*n* = 5). Statistics: pairwise permutation test with Benjamini-Hochberg correction for multiple comparison. Significant differences (*p* < 0.05) are indicated by differing letters.

SUPPLEMENTARY FIGURE S3 Neuronal differentiation efficiencies between the different iPSC lines. WT and Ig-Ctrl cells originating from iPSC#1 differentiate the most efficient. Cells originating from iPSC#2 reach a max average differentiation efficiency of 40%.

SUPPLEMENTARY FIGURE S4 Neuron-like cells from both iPSC lines used in this study were analysed for cell survival responses on DMSO treatment (rotenone was dissolved in DMSO). **(A–C)** Cell survival of iPSC-derived neurons originating from iPSC#1. Exposure to Carrier (same concentration of DMSO as rotenone-containing treatments) had no impact on the survival of WT **(A)** and Ig-Ctrl **(B)** neurons but reduced survival of KO neurons **(C)**, however survival is higher than in rotenone treated cells. **(D–F)** Cell survival of iPSC-derived neurons originating from iPSC#2. Exposure to Carrier (same concentration of DMSO as rotenone-containing treatments) had no impact on the survival of WT, Ig-Ctrl and KO neurons. However, cell survival of Ig-Ctrl and KO cells was not significantly different to rotenone treated cells. Statistics: pairwise permutation test with Benjamini-Hochberg correction for multiple comparison. Significant differences (*p* < 0.05) are indicated by differing letters.
