# Supplementary figures and images for: The cytokine receptor CRLF3 is a human neuroprotective EV-3 (Epo) receptor
Source: Front Mol Neurosci. 2023 Apr 6;16:1154509. doi: 10.3389/fnmol.2023.1154509 (PMC10165946; doi:10.3389/fnmol.2023.1154509)

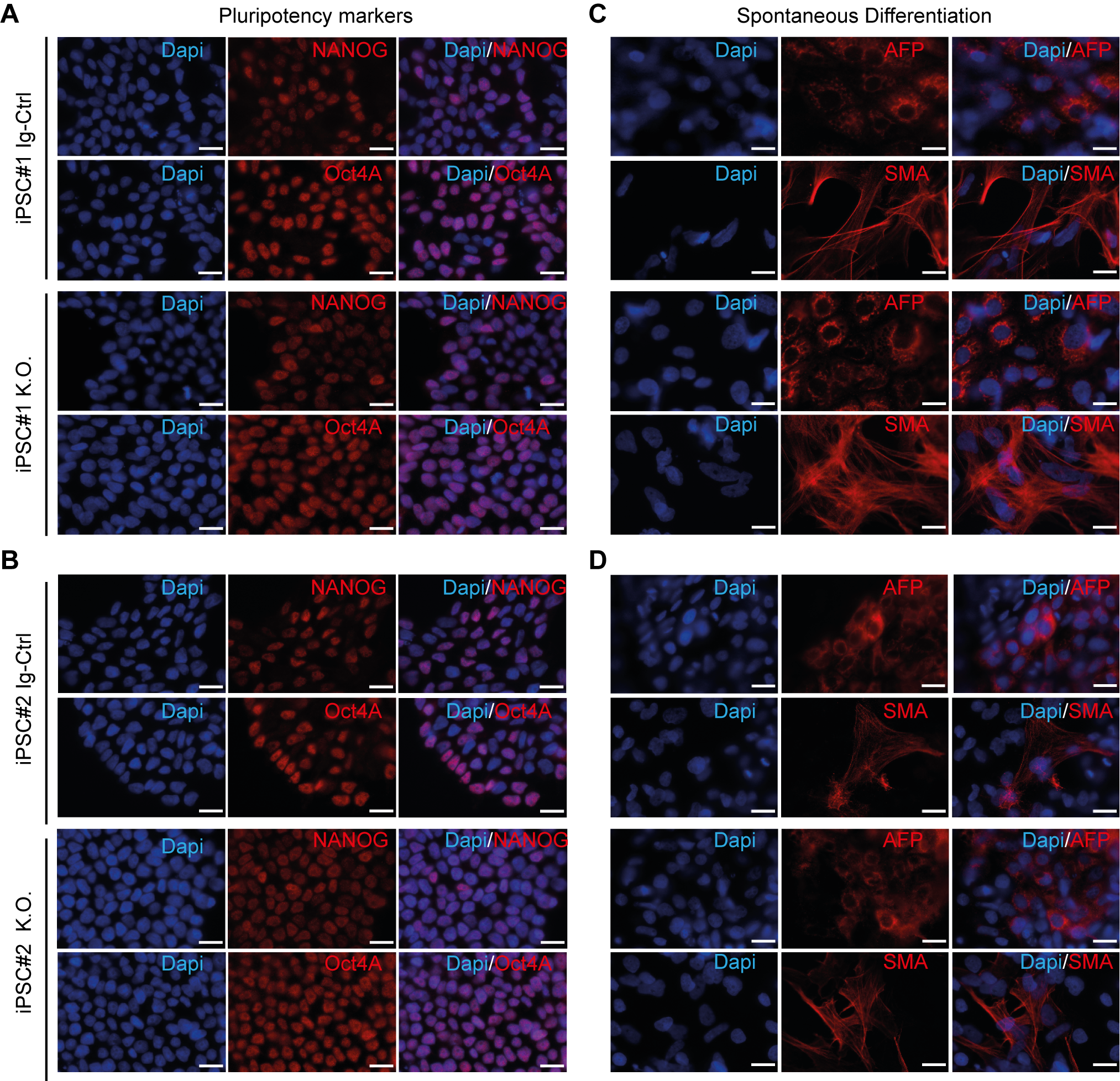

Supplement: SUPPLEMENTARY FIGURE S1 — Characterization of newly generated iPSC lines. For both line 1 and 2, Ig-Ctrl and CRLF3 KO cells were examined to ensure full pluripotency. (A,B) iPSC of all four lines were stained for two characteristic pluripotency markers. All cells show clear nuclear staining for Nanog and OCT4A. (C,D) Ig-Ctrl and KO cells of both lines were used to form embryoid bodies and allow spontaneous differentiation. Cells were stained for characteristic markers of the three germ layers. All four lines generate immunopositive cells for alpha-fetoprotein (AFP) representing endodermal tissue and smooth muscle actin (SMA) representing mesoderm. ß-III-tubulin stainings for ectodermal differentiation are presented in Figure 2 of the main manuscript. Scale bars: 20 µM. [file Image_1.TIF]

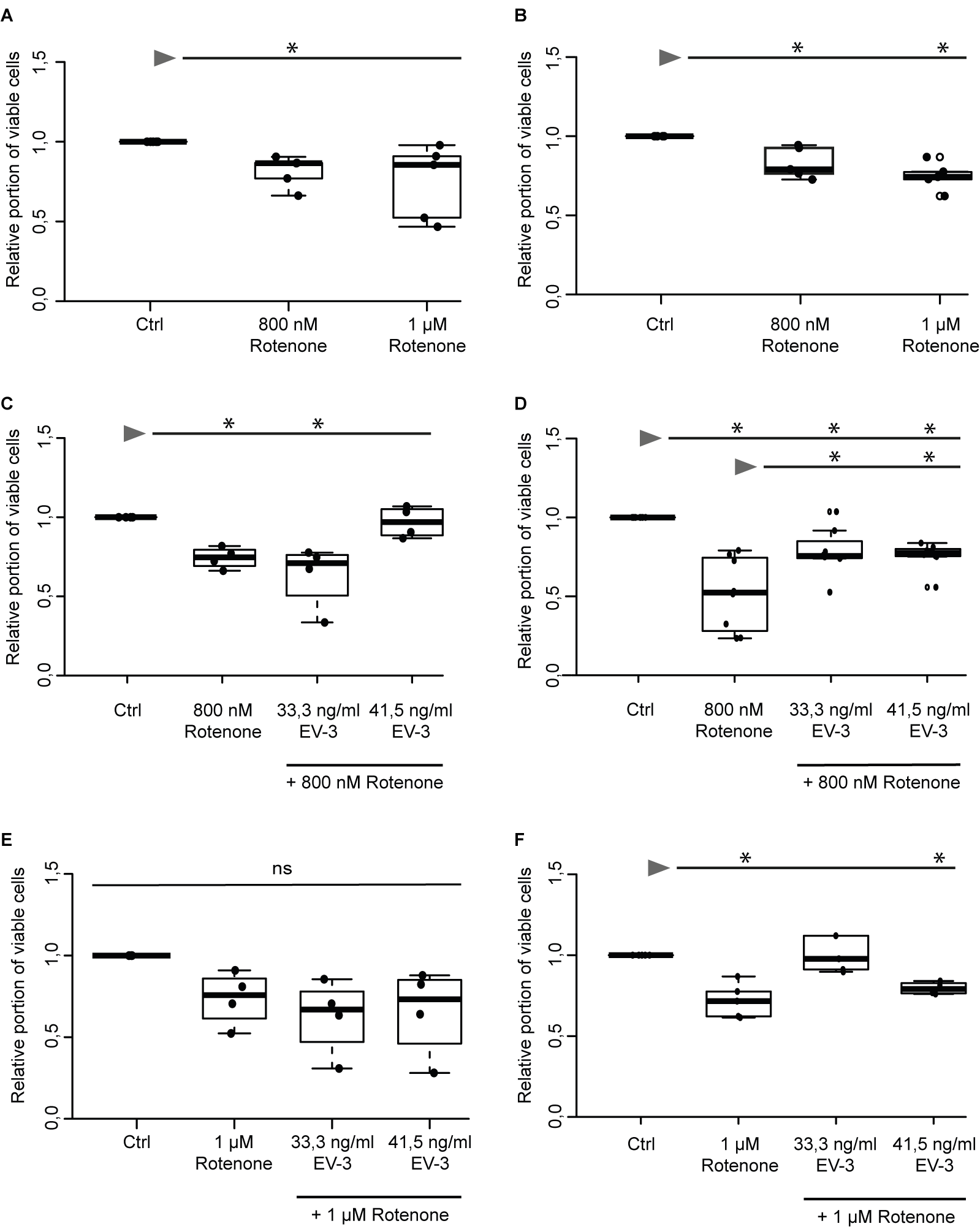

Supplement: SUPPLEMENTARY FIGURE S2 — Survival assays with iPSC-derived neurons. iPSC lines 1 and 2 (left and right column respectively) were differentiated into neurons and subsequently (on day 30 of differentiation) treated with either rotenone alone or rotenone and EV-3. (A1,A2) iPSC-derived neurons were treated with either 800 nM or 1 µM rotenone for 18 h. Cell survival was measured using FACS. Survival in both cell lines is significantly reduced when cells receive 800 nM rotenone. 1 µM rotenone significantly decreases cell survival in line 2, however not in line 1. n = 5 for both lines. (B1) Cell death induced by 800 nM rotenone is unaffected by 33.3 ng/ml EV-3 but completely prevented by 41.5 ng/ml EV-3 in iPSC-derived neurons of line 1 (n = 4). (B2) Cell death induced by 800 nM rotenone is partially prevented by both concentrations of EV-3 in line 2 iPSC-derived neurons (n = 8). (C1) Cell death induced by 1 µM rotenone is unaffected by EV-3 in line 1 iPSC-derived neurons (n = 4). (C2) Cell death induced by 1 µM rotenone is unaffected by 41.5 ng/ml EV-3 but partially reduced by 33.3 ng/ml EV-3 (n = 5). Statistics: pairwise permutation test with Benjamini-Hochberg correction for multiple comparison. Significant differences (p < 0.05) are indicated by differing letters. [file Image_2.TIF]

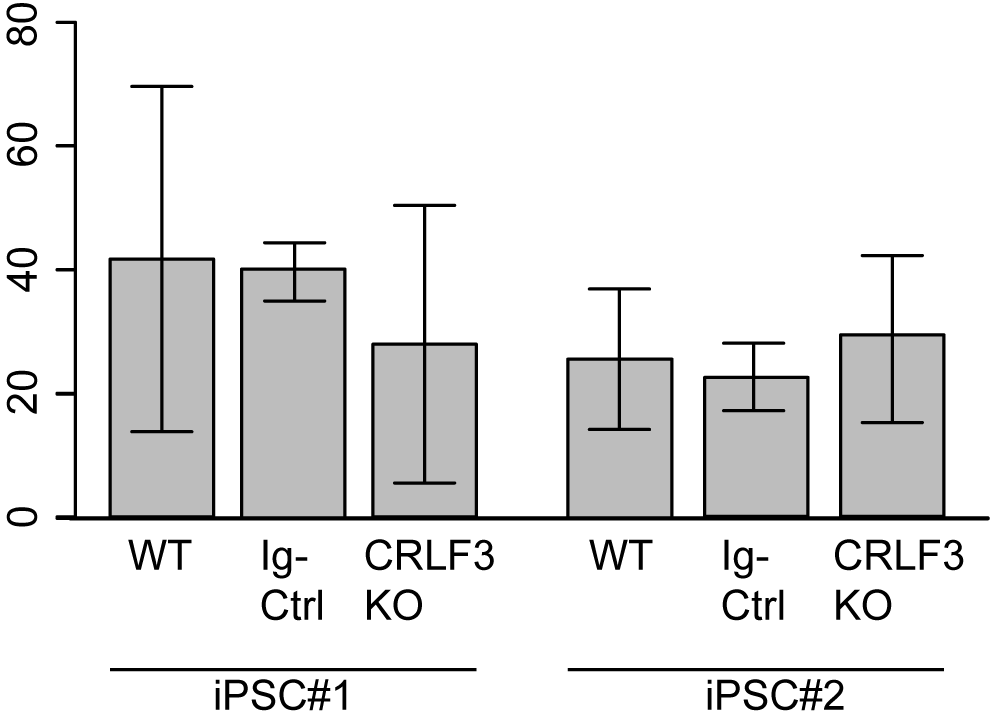

Supplement: SUPPLEMENTARY FIGURE S3 — Neuronal differentiation efficiencies between the different iPSC lines. WT and Ig-Ctrl cells originating from iPSC#1 differentiate the most efficient. Cells originating from iPSC#2 reach a max average differentiation efficiency of 40%. [file Image_3.TIF]

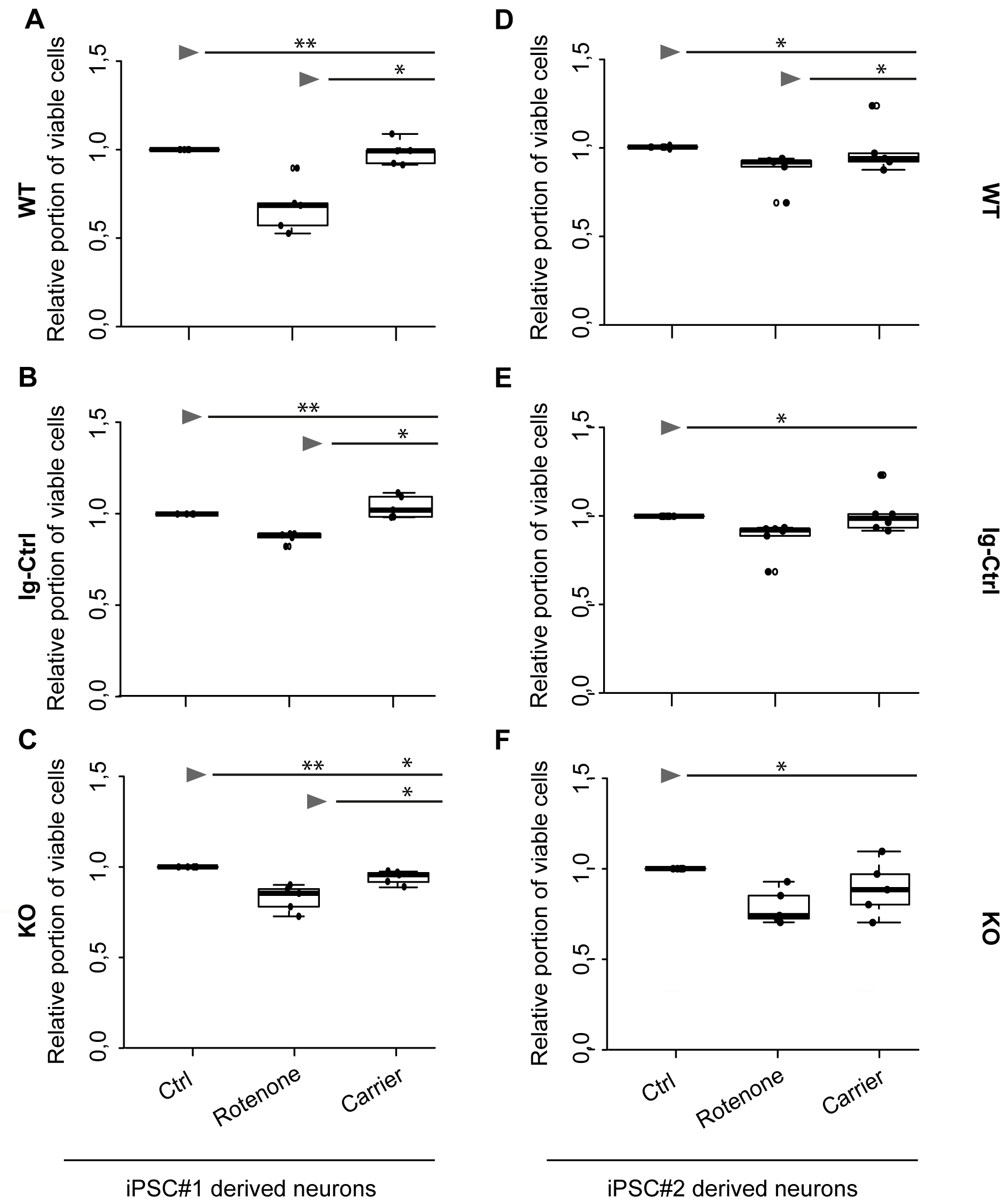

Supplement: SUPPLEMENTARY FIGURE S4 — Neuron-like cells from both iPSC lines used in this study were analysed for cell survival responses on DMSO treatment (rotenone was dissolved in DMSO). (A–C) Cell survival of iPSC-derived neurons originating from iPSC#1. Exposure to Carrier (same concentration of DMSO as rotenone-containing treatments) had no impact on the survival of WT (A) and Ig-Ctrl (B) neurons but reduced survival of KO neurons (C), however survival is higher than in rotenone treated cells. (D–F) Cell survival of iPSC-derived neurons originating from iPSC#2. Exposure to Carrier (same concentration of DMSO as rotenone-containing treatments) had no impact on the survival of WT, Ig-Ctrl and KO neurons. However, cell survival of Ig-Ctrl and KO cells was not significantly different to rotenone treated cells. Statistics: pairwise permutation test with Benjamini-Hochberg correction for multiple comparison. Significant differences (p < 0.05) are indicated by differing letters. [file Image_4.TIF]
